# Lithium-Directed Transformation of Amorphous Iridium (Oxy)hydroxides To Produce Active Water Oxidation Catalysts

**DOI:** 10.1021/jacs.2c13567

**Published:** 2023-03-09

**Authors:** Jonathan Ruiz Esquius, David J. Morgan, Gerardo Algara Siller, Diego Gianolio, Matteo Aramini, Leopold Lahn, Olga Kasian, Simon A. Kondrat, Robert Schlögl, Graham J. Hutchings, Rosa Arrigo, Simon J. Freakley

**Affiliations:** † Max Planck-Cardiff Centre on the Fundamentals of Heterogeneous Catalysis FUNCAT, Cardiff Catalysis Institute, School of Chemistry, Cardiff University, Main Building, Park Place, Cardiff CF10 3AT, U.K.; ‡ International Iberian Nanotechnology Laboratory, Av. Mestre José Veiga, Braga 4715-330, Portugal; § Department of Inorganic Chemistry, Fritz Haber-Institut der Max-Planck-Gesellschaft, 14195 Berlin, Germany; ∥ Diamond Light Source Ltd, Harwell Science and Innovation Campus, Fermi Avenue, Didcot OX11 0DE, U.K.; ⊥ Helmholtz Institut Erlangen-Nürnberg, Helmholtz-Zentrum Berlin GmbH, Cauerstr. 1, 91058 Erlangen, Germany; ∇ Department of Materials Science and Engineering, Friedrich-Alexander-Universität Erlangen-Nürnberg, 91058 Erlangen, Germany; ○ Department of Chemistry, Loughborough University, Epinal Way, Loughborough, Leicestershire LE11 3TU, U.K.; ◆ Department of Heterogeneous Reactions, Max Planck Institute for Chemical Energy Conversion, 45470 Mulheim an der Ruhr, Germany; ¶ School of Science, Engineering and Environment, University of Salford, Manchester M5 4WT, U.K.; & Department of Chemistry, University of Bath, Claverton Down, Bath BA2 2AY, U.K.

## Abstract

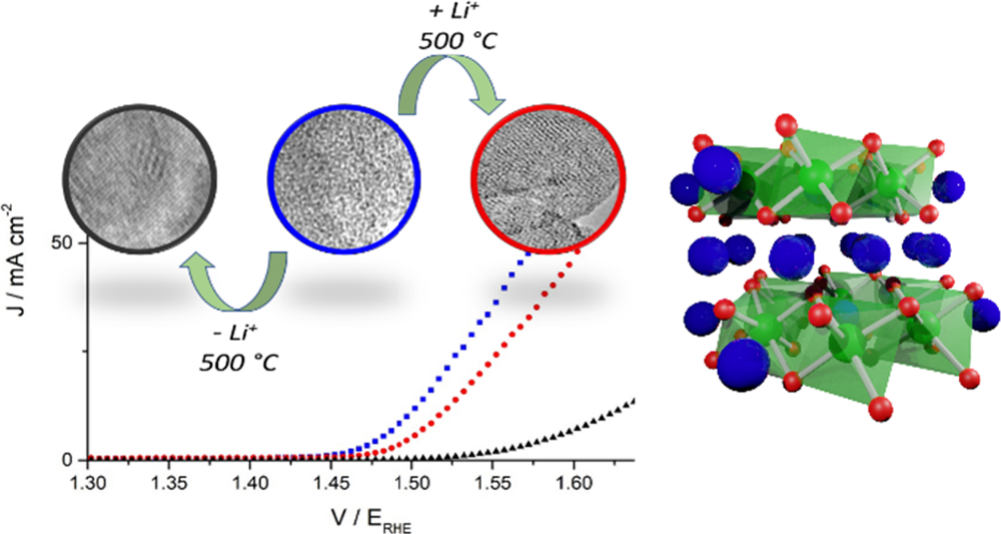

The oxygen evolution reaction (OER) is crucial to future energy systems based on water electrolysis. Iridium oxides are promising catalysts due to their resistance to corrosion under acidic and oxidizing conditions. Highly active iridium (oxy)hydroxides prepared using alkali metal bases transform into low activity rutile IrO_2_ at elevated temperatures (>350 °C) during catalyst/electrode preparation. Depending on the residual amount of alkali metals, we now show that this transformation can result in *either* rutile IrO_2_ or nano-crystalline Li-intercalated IrO_*x*_. While the transition to rutile results in poor activity, the Li-intercalated IrO_*x*_ has comparative activity and improved stability when compared to the highly active amorphous material despite being treated at 500 °C. This highly active nanocrystalline form of lithium iridate could be more resistant to industrial procedures to produce PEM membranes and provide a route to stabilize the high populations of redox active sites of amorphous iridium (oxy)hydroxides.

## Introduction

Large-scale water electrolysis is key to the renewable chemical industry. Catalysts for the oxygen evolution reaction (OER) are often limited by instability under anodic potential in acidic electrolytes. Iridium oxides show promise under these conditions with comparatively limited corrosion compared to other catalysts such as RuO_2_.^1−3^ Various morphologies have been investigated to maximize activity and develop a fundamental understanding of the catalytic process. Metallic Ir nanoparticles have been studied under OER conditions and shown to form amorphous iridium(oxy)hydroxide (Ir(O)_*x*_(OH)_*y*_) shells which are the active form of the catalyst.^4,5^ Nanoparticulate and thin film Ir(O)_*x*_(OH)_*y*_ outperforms crystalline rutile IrO_2_ in terms of reduced overpotential (η)^6,7^ which has been associated with higher structural flexibility,^8,9^ coexistence of Ir(III)/Ir(IV),^10^ and formation of electrophilic O^–^ sites under OER conditions.^11,12^ Geiger et al. reported that annealing amorphous Ir(O)_*x*_(OH)_*y*_ films results in rutile IrO_2_ (r-IrO_2_) with reduced intrinsic activity, suggesting a fine balance between activity, stability, and structure of the catalysts.^10,13^ X-ray absorption spectroscopy (XAS) has been extensively applied to study the electronic structure of catalysts *in situ* and suggests that Ir(IV) oxides form higher oxidation-state species under OER conditions.^14^ This is supported by *operando* X-ray photoelectron spectroscopy (XPS) which revealed oxidized Ir species bound to electron-deficient oxygen.^11^ The abundance of this oxyl species was linearly correlated with charge transfer suggesting formation via an oxidation/deprotonation of surface hydroxyl groups. Less crystalline Ir(O)_*x*_(OH)_*y*_ therefore enables an abundance of hydroxyl groups with increased structural flexibility that may easily coordinate water providing a path for O–O bond formation.

Gao et al. recently reported amorphous Li^+^-doped IrO_*x*_ catalysts (η = 270 mV at 10 mA cm^–2^) with high activity suggested to originate from flexible IrO_6_ octahedra resulting from Li^+^ incorporation not present in rutile IrO_2_.^15^ Willinger et al. studied amorphous Ir(O )_*x*_(OH)_*y*_ and similarly proposed flexible hollandite structural motifs as active sites.^16^ Alkali-doped hollandite structures can be prepared under harsh conditions; Sun et al. synthesized K_0.25_IrO_2_ from IrCl_3_/K_2_CO_3_ by annealing in air (600 °C, 6 h), and distorted IrO_6_ octahedra were suggested to increase activity compared to stable rutile IrO_2_.^17^ We recently reported an amorphous IrO_*x*_ prepared via hydrothermal synthesis using Li_2_CO_3_ which showed high activity (η = 250 mV at 10 mA cm^–2^) in comparison to a commercial hydrated IrO_*x*_ with a comparable Ir(III):Ir(IV) ratio, geometric surface area, and surface composition to the as-prepared material.^18^ The higher intrinsic activity, when normalized to active electrochemical surface area (ECSA), suggested a possible promotional effect of residual alkali metal ions, in this case Li^+^.

We now show that starting from amorphous Ir(O)_*x*_(OH)_*y*_, it is possible to form *either* rutile or Li-intercalated iridium oxide during thermal treatments depending on the level of residual Li^+^. We find that this Li-intercalated iridium oxide shows comparable activity and increased stability compared to the amorphous material despite being treated at 500 °C—breaking the relationship between thermal treatment and deactivation of Ir(O)_*x*_(OH)_*y*_. Operando spectroscopy reveals mechanistic insights, and the stability of this material provides a framework for the design of improved OER anode systems.

## Results and Discussion

### Structural Characterization of Iridium Oxides

**Figure 1:**
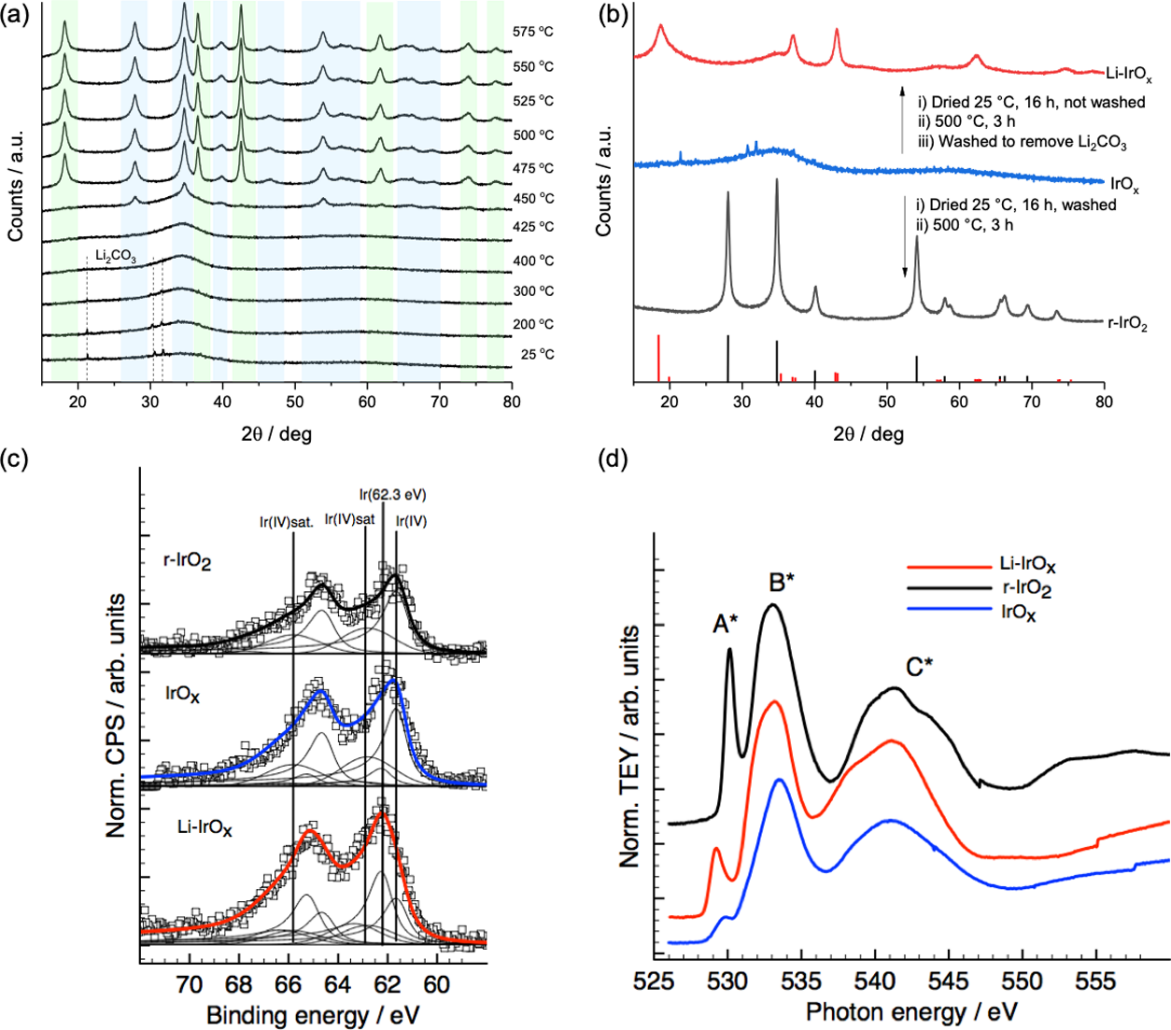
(a) *In situ* XRD pattern of as-prepared IrO_*x*_-Li_2_CO_3_ under flowing air held for 5 min at each temperature before recording a diffraction pattern. Blue regions indicate rutile IrO_2_ reflections and green regions represent Li-IrO_*x*_. (b) XRD pattern of samples prepared by divergent preparation methods to control the residual Li_2_CO_3_. Black reference pattern rutile IrO_2_ [ICSD-56009], red reference pattern β-Li_2_IrO_3_ [ICSD-246025]. (c) Fitted Ir 4f XPS spectra relative to the samples as indicated, measured in UHV and at an electron kinetic energy (KE) of 200 eV (left). (d) Total electron Yield (TEY) O K-edge NEXAFS spectra measured for IrO_*x*_, Li-IrO_*x*_, and r-IrO_2_.

The synthesis of iridium-oxyhydroxide (IrO_*x*_) using Li_2_CO_3_ as a precipitating base was reported by our group as the basis for this study.^18^ X-ray diffraction (XRD, Figure 1a) of the freshly prepared material shows broad features at 35°, typical of amorphous IrO_*x*_ and low-intensity diffraction of residual Li_2_CO_3_ [ICSD-16713]. *In situ* diffraction during heat treatments shows rutile IrO_2_ [ICSD-56009] forms >425 °C consistent with the crystallization of amorphous IrO_*x*_. The diffraction pattern of Li_2_CO_3_ is no longer observed, suggesting reaction or decomposition at lower-than-expected temperatures compared to the literature (∼727 °C).^19^ An additional diffraction pattern distinct to rutile IrO_2_ developed above 450 °C, which is highlighted in green in Figure 1a. To probe the effect of residual Li_2_CO_3_, two further samples were prepared from the same IrO_*x*_: (i) washed to remove all Li_2_CO_3_ (determined by the absence of the Li_2_CO_3_ diffraction pattern) and (ii) not washed to retain Li_2_CO_3_ after filtration and drying. Both were heated in static air (500 °C 3 h) and washed to remove residual Li_2_CO_3_. XRD (Figure 1b) shows distinctive phase transitions depending on the presence of Li_2_CO_3_. The Li^+^-free IrO_*x*_ crystallizes into rutile IrO_2_ (r-IrO_2_) without any contaminant phase, confirmed by comparison to commercial IrO_2_ (Figure S1). The sample with excess Li_2_CO_3_ crystallizes exclusively into a different phase (Li-IrO_*x*_) with a crystallite size of ∼11 nm; a small residual broad feature is consistent with some remaining amorphous material.

Lithium iridates are typically prepared by a high-temperature solid-state reaction between IrO_2_/Ir black and Li_2_CO_3_.^20−22^ Grimaud prepared β-Li_2_IrO_3_ by reacting Ir/IrO_2_ and Li_2_CO_3_ at 1080 °C for 30 h.^23^ Chan prepared similar Li-rich Li_*n*_IrO_*x*_ by reacting IrO_2_ and Li_2_CO_3_ at 950 °C for 2 days.^24^ Both β-Li_2_IrO_3_ and α-Li_2_IrO_3_ contain layered structures of Ir-oxo sheets and intercalated Li^+^ characterized by XRD between 18 and 20°. This can vary from ∼18.2° for high Li^+^ contents to 20° for low Li^+^ or Li/H^+^ intercalation corresponding to the IrO_6_ sheet inter-planar distance.^25^ The obtained new diffraction pattern largely aligns with the β-Li_2_IrO_3_ phase reported by O’Malley et al. who prepared samples by heating Li_2_CO_3_ and Ir metal at 750 °C 12 h before the increasing temperature to 1050 °C [ICSD-246025].^22^ The nanocrystalline nature of our material means that low-intensity X-ray reflections of highly crystalline samples are likely below the detection limits. The observed principle reflection (18.3°) suggests that the layered Li-IrO_*x*_ has 0.48 nm spacing between Ir-oxo planes consistent with Li stoichiometry close to 2.^26^

XPS spectra of the Ir 4f levels (Figure 1c) were fitted considering an asymmetric main peak and shake-up satellite +1 eV to the main line.^10^ A component (61.8 eV) consistent with crystalline rutile IrO_2_ was observed for the synthesized r-IrO_2_.^7^ An additional component (62.3 eV) appears with associated satellite for the IrO_*x*_ material; this is the main component for the Li-IrO_*x*_ material and was reported for many amorphous iridium-oxyhydroxides *in situ* during OER.^6,27^*In situ* XPS studies suggest that Ir-oxide nanoparticles have surface oxidation states below Ir(IV) when terminal H_2_O/OH species are chemisorbed with an Ir 4f binding energy of 62.3 eV. This suggests that the surfaces of both IrO_*x*_ and particularly Li-IrO_*x*_ are highly hydrated/hydroxylated rather than oxide-terminated.^28^ Changes in the Ir oxidation state are mirrored by small binding energy shifts which are also sensitive to surface orientation/termination, making assignment of the formal surface oxidation state challenging, especially for a distribution of species. Surface oxygen species were investigated by O K-edge NEXAFS (Figure 1d) and characterized by two sharp resonances, A* and B*, assigned to O 1s excitation into hybridized O 2p-Ir 5d *t*_2g_ and e_g_ states, in addition to a higher energy resonance (C*) from excitation into O 2p-Ir 6s-p hybridized states, respectively. A high ratio of A*:B* is characteristic of high M–O covalency and the degree of hybridization between O 2p and *t*_2g_ orbitals of the M–O bond.^29^ The r-IrO_2_ spectrum (Figure 1d) presents strong resonances at 530 and 533 eV in agreement with literature data for O^2–^ species.^6^ A significant decrease in the A* intensity for IrO_*x*_ indicates lower degree of hybridization, consistent with a higher ionic character. Moreover, this resonance appears split into two components with the additional component at 529 eV. Similarly, Li-IrO_*x*_ has a less intense A* resonance, consistent with Li_2_IrO_3_ systems, and shifts to lower energy (∼529 eV).^30^ This pre-edge resonance was associated with electron hole states in hybridized Ir–O orbitals due to electrophilic oxygen species (oxyl species) which were suggested as active O species for nucleophilic attack by H_2_O to form O–O bonds.^9^ The presence of a hole in the oxygen valence band generates an intermediate valence ground state, with consequent appearance of extra features in the O K-edge spectra due to multielectron configuration in the excited final state; the apparent splitting of resonance B* for IrO_*x*_ and Li-IrO_*x*_ could also be qualitatively explained by the presence of these species,^31^ which are more pronounced for the Li-IrO_*x*_ system. Inspection of the resonance C* region unveils peculiar structural characteristics of Li-IrO_*x*_ compared to that of r-IrO_2_. Using a simple molecular orbital description, the broad resonance contains both the transition to the 3*a*_1g_ level (Ir 6s; O 2pσ) and the 4*t*_1u_ (Ir 6p; O 2pπ) level. In r-IrO_2_, the decrease of the Ir–Ir distance in the direction perpendicular to the edges shared by the octahedra is associated with a degree of distortion of the Ir-O_6_ octahedra.^32^ This Ir–O distance is reflected in the position of the C* resonances, whose peak maximum appears at approximately 539, 541, and 545 eV in r-IrO_2_. A weaker interaction between Ir 6sp and O 2p (less dense structure) leads to a higher energy position of the bonding orbitals, thus a lower energy position of the antibonding orbitals. In Li-IrO_*x*_, the C* resonances are found with maximum at 538 and 541 eV, indicating longer Ir–O bonds, whereas the distortion typical of rutile-type IrO_2_ is no longer observed. On IrO_*x*_, this resonance is broader, indicating a larger distribution of Ir–O bond lengths.

**Figure 2:**
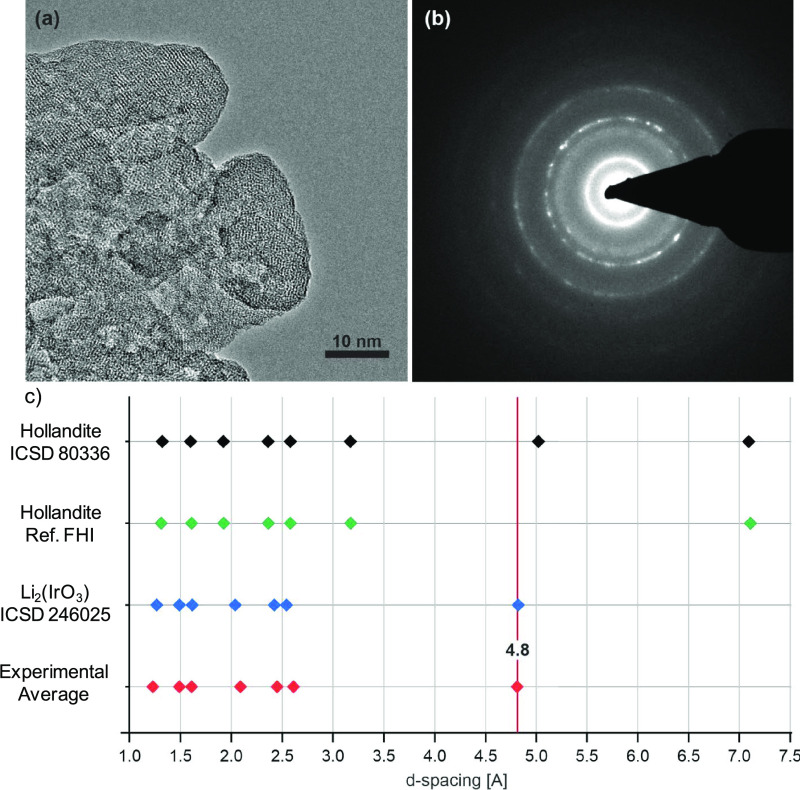
Electron microscopy characterization of Li-IrO_*x*_. (a) High-resolution TEM image showing nanocrystalline domains. (b) Electron diffraction pattern of the heated sample corroborating that the sample is polycrystalline, (c) comparison between simulated hollandite and lithium iridate (β-Li_2_IrO_3_) as well as experimental hollandite (FHI)^16^ and the average *d*-spacings taken from Li-IrO_*x*_.

**Figure 3:**
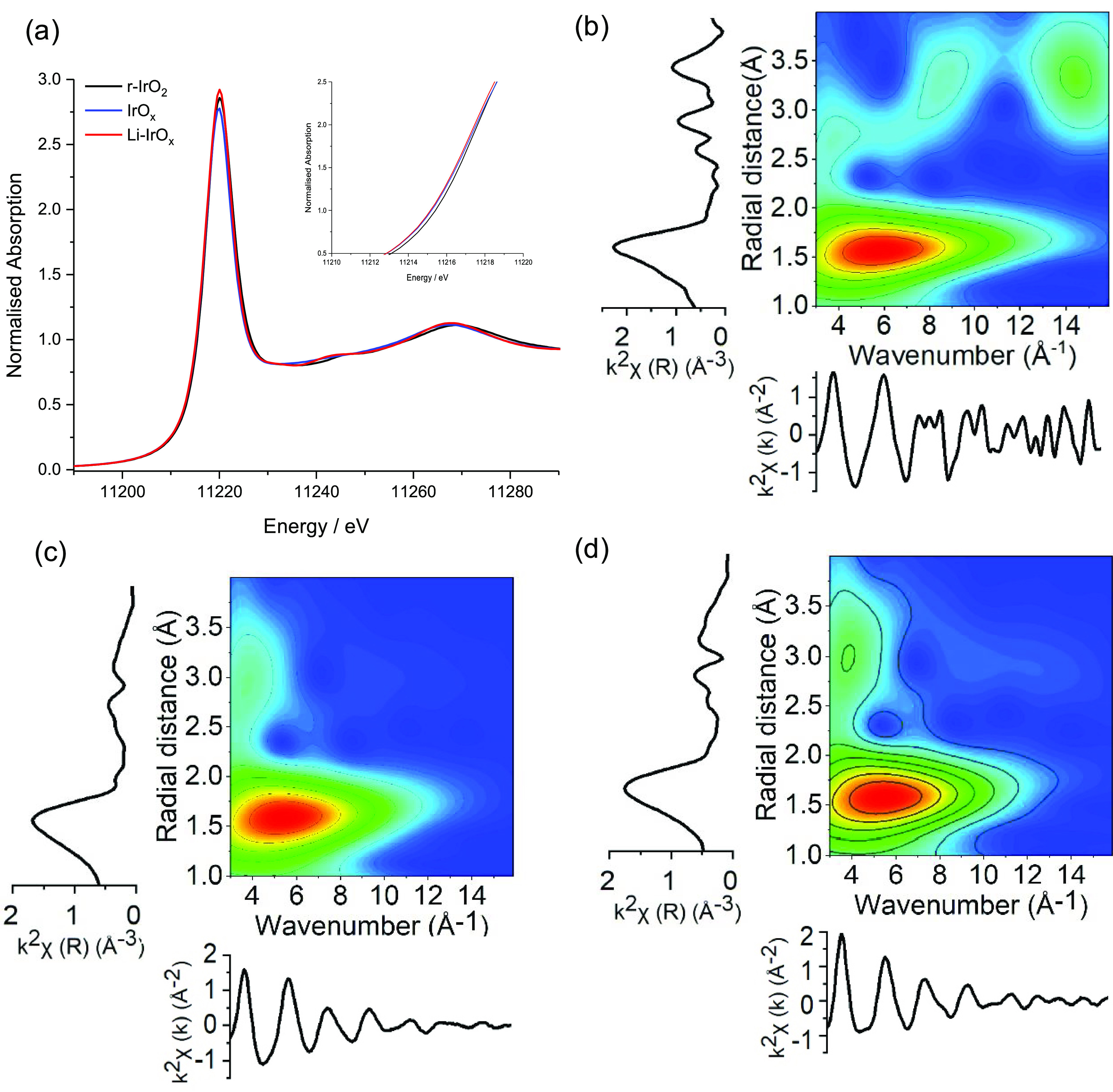
(a) Transmission Ir-L_3_-edge XANES and wavelet EXAFS plots, with corresponding *k*^2^ and FT magnitudes, for (b) r-IrO_2_, (c) IrO_*x*_, and (d) Li-IrO_*x*_.

High-resolution aberration-corrected transmission electron microscopy (TEM) shows that IrO_*x*_ appears, as expected, amorphous (Figure S2), while Li-IrO_*x*_ is polycrystalline with nanocrystalline domains (Figure 2). The Li-IrO_*x*_ sample shows electron diffraction at *d*-spacing corresponding to those of lithium iridate (β-Li_2_IrO_3_) (ICSD-246025) consistent with XRD. The electron diffraction at multiple spots clearly shows that the material does not consist of rutile or alkali metal hollandite phases of IrO_2_. The samples were studied by XAS at the Ir L_3_-edge where interpretation of the absorption edge is complicated by a white line (WL) which is sensitive to symmetry, ligand environment, and oxidation state.^33^Figure 3a shows that r-IrO_2_, IrO_*x*_, and Li-IrO_*x*_ have similar WL positions (11,219.8–11,220.0 eV) defined by the minimum in the second derivative. Comparison of Ir^0^ (5d^7^), IrCl_3_ (5d^6^), and IrO_2_ (5d^5^) (Figure S3) suggests a mixed valance Ir(IV)/Ir(III) species in the disordered IrO_*x*_ material supported qualitatively by the reduced WL height as a direct probe of 5d occupancy.^18^

Extended X-ray absorption fine structure (EXAFS) spectra and wavelet transformations are shown in Figure 3; fitting parameters and suggested fits are shown in Tables 1 and S1 and Figures S4–5. r-IrO_2_ was satisfactorily modeled with a first Ir–O_6_ shell and second Ir–Ir shell.^14^ The Ir–O path at 1.97(2) Å with a coordination number (CN) of 6.1(1.6) was consistent with Ir(IV) oxide species.^34^ Ir–Ir_1_ had a path length of 3.15(2) Å and a CN of 3.0(1.5) and Ir–Ir_2_ at 3.55(1) Å and a CN of 6.8(1.2). Including Ir–O multiple scattering paths resulted in unrealistic 2σ^2^ values. The wavelet transformation shows a strong feature centered at low *R* (Δ*k* 3–13 Å^–1^ and Δ*R* 1–2 Å) associated with the first Ir–O shell. The features at Δ*k* 3–8 Å^–1^ and Δ*R* 2.5–3.0 Å are attributed to subsequent Ir–O shells and multiple scattering paths. The split feature at high Δ*k* 9–16 Å^–1^ and Δ*R* 3.0–3.75 Å is due to the two heavy scattering Ir–Ir paths. Therefore, EXAFS analysis of r-IrO_2_ confirms findings from XRD of an ordered rutile structured material. Contrasting IrO_*x*_ and Li-IrO_*x*_ with r-IrO_2_ wavelet analysis, the first Ir–O_6_ shell remains relatively consistent with a diminished contribution from the heavy scattering Ir–Ir paths. An additional feature (Δ*k* 3–6 Å^–1^, Δ*R* 2.75–3.5 Å) not seen in r-IrO_2_ is observed for IrO_*x*_ and with greater intensity in Li-IrO_*x*_, indicating the presence of another light scatterer such as a different O species or Li. The data were fitted with a model of layered 2D edge-sharing [IrO_6_], derived from the crystal structure of β-Li_2_IrO_3_ (ICSD-246025), including two simplified Ir–O shells (using a single averaged Ir–O distance vs the two distorted [IrO_6_] environments within the β-Li_2_IrO_3_ crystal structure) and a single Ir–Ir path.^22^ The CN of the first Ir–O path was floated, while all other path degeneracies were fixed. Given the challenges in accurately modeling Li scatterers, the fitting model used omitted these paths, although models including the Ir–Li paths are included in the Supporting Information (Table S1). IrO_*x*_ is defined by a first shell [IrO_6_] octahedron (Ir–O of 2.013(4) Å, CN 5.9(3)); elongated Ir–O bonds are consistent with increased structural disorder (higher 2σ^2^) relative to r-IrO_2_ and reduced Ir species.^35^ The second shell Ir–Ir (3.14(2) Å, CN = 6) and a high 2σ^2^ support layered edge-sharing Ir–O_6_ octahedra with a longer Ir–Ir distance than crystalline β-Li_2_IrO_3_ (2.98 Å), suggesting increased structural disorder. No improvement in fitting was observed upon the inclusion of Li scattering paths, showing that EXAFS provides no evidence for the presence of ordered Li within the sample. A comparable fit was determined for Li-IrO_*x*_, with a sightly reduced Ir–O first shell distance of 2.000(6) Å and an Ir–Ir distance of 3.09(2) Å, coupled with a reduction in the latter paths 2σ^2^, indicating an increased structural order, further demonstrated by a visible XRD pattern. The inclusion of Li scattering paths (Table S1) did improve fitting results for Li-IrO_*x*_, although given the challenge of fitting the extremely light Li scatters, the result is validated only by corroboration from the clear evidence of the layered structure by X-ray and electron diffraction. Therefore, EXAFS supports findings of the formation of a layered Li-IrO_*x*_ material at relatively mild temperatures compared to other reported synthetic procedures. The comparison of this structure to that of rutile IrO_2_ based on the cited ISCD entries is shown in Figure S6.

**Table 1:** EXAFS Model Fitting Parameters for Fresh r-IrO_2_, IrO_*x*_, Li-IrO_*x*_, and IrCl_3_ from Data Measured at the Ir-L_3_-Edge

sample	scattering path	CN	*R* (Å)	2σ^2^ (Å^2^)	*S*_o_^2^	*E*_f_ (eV)	*R*_factor_ + (χ^2^)
IrCl_3_	Ir–Cl_1_	6^a^	2.33(1)	0.003(1)	0.79	7.5(3)	0.018
r-IrO_2_	Ir–O	6.1(1.6)	1.97(2)	0.002(10	0.79^a^	11.8(6)	0.04 (2830)
	Ir–Ir_1_	3.0(1.5)	3.14(2)	0.004(2)			
	Ir–Ir_2_	6.8(1.2)	3.55(1)	0.003(1)			
IrO_*x*_	Ir–O	5.9(4)	2.013(4)	0.005(1)		12.5(5)	0.010 (2961)
	Ir–Ir	6^a^	3.14(2)	0.013(2)	0.79^a^		
	Ir–O_2_	6^a^	3.64(2)	0.007 (2)			
Li-IrO_*x*_	Ir–O	6.3(5)	2.000(6)	0.005(1)		12(1)	0.028 (2128)
	Ir–Ir_1_	6^a^	3.09(2)	0.011(2)	0.79^a^		
	Ir–O_2_	6^a^	3.62(3)	0.006(3)			

a Ir–Cl CN fixed at 6 to determine *S*_o_^2^ at a value of 0.79.

### Electrocatalytic Data

Lithium iridates prepared under harsh conditions (950 °C, 2 days) have been studied electrochemically to probe charging/discharging of Li^+^ ions above 2 V_RHE_.^24^ The study suggests that at higher potentials, the charge capacity was predominantly attributed to oxidation of O^2–^ ions drawing similarity to OER mechanisms. Furthermore, McCalla et al. observed O–O peroxo dimers in similar layered oxide-type Li batteries.^36^ Tarascon extended the study of lithium iridates suggesting that the open structure allows water to penetrate between the MO_2_ planes rendering “bulk” (O_2_)^*n*−^ species active for the OER by Li/H exchange.^37^ Pearce et al. synthesized highly crystalline β-Li_2_IrO_3_ prepared from IrO_2_ and Li_2_CO_3_ at 1080 °C for 30 h and demonstrated that it was stable to dissolution under acidic conditions and under polarization is active towards the OER while undergoing Li/H^+^ exchange.^25^

**Figure 4:**
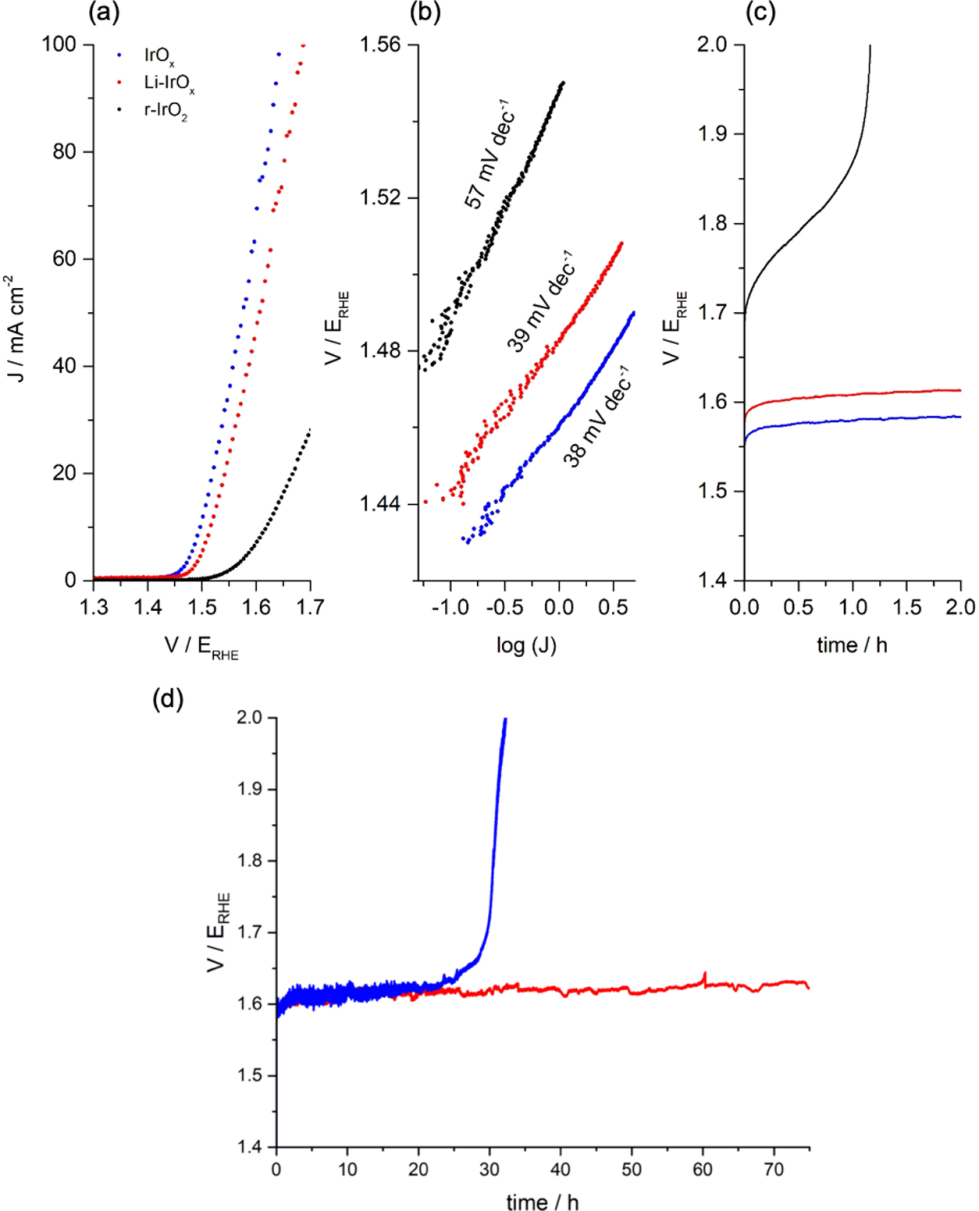
IrO_*x*_, r-IrO_2_, and Li-IrO_*x*_ (a) activity (LSV, 1.2–1.7 V_RHE_, 5 mV s^–1^). (b) Tafel slope obtained from semi-steady-state conditions (LSV, 0.5 mV s^–1^). (c) Long-term stability (CP, 10 mA cm^–2^, 2 h) and (d) (CP, 10 mA cm^–2^, 75 h) toward OER in a 0.1 M HClO_4_ electrolyte.

The OER activity of IrO_*x*_, r-IrO_2_, and Li-IrO_*x*_ in a 0.1 M HClO_4_ electrolyte was assessed by LSV (1.2–1.7 V_RHE_, 5 mV s^–1^) without any pre-activation treatment (Figure 4a). r-IrO_2_, Li-IrO_*x*_, and IrO_*x*_ achieved 10 mA cm^–2^ at an overpotential (η) of 390, 290, and 270 mV, respectively. The synthesized Li-IrO_*x*_ shows comparable activity to both IrO_*x*_ and commercially available IrO_2_·2HO (Premion Alfa Asear) in terms of the potential required to reach 10 mA cm^–2^ (IrO_*x*_ −1.50 V, Li-IrO_*x*_ −1.51 V, IrO_2_·H_2_O −1.52 V—despite being thermally treated at 500 °C. Similar electrochemically active surface areas (ECSAs) were obtained for IrO_*x*_ (0.005 mF cm^2^) and Li-IrO_*x*_ (0.006 mF cm^2^) by normalizing the double-layer capacitance by the specific capacitance (Figure S7).^38^ Tafel slopes (Figure 4b) for IrO_*x*_ (38 mV dec^–1^) and Li-IrO_*x*_ (39 mV dec^–1^) agreed with previous results reported for (oxy)hydroxides (ca. 40 mV dec^–1^), which suggests a similar dependence of the reaction rate on potential.^15,39,40^ However, a higher Tafel slope for r-IrO_2_ (57 mV dec^–1^) was in agreement with literature (ca. 60 mV dec^–1^) and slower kinetics.^41,42^ Comparable onset potentials between Li-IrO_*x*_ and IrO_*x*_, similar ECSA and Tafel slopes indicate that the layered nanocrystalline Li-IrO_*x*_ has similar electrochemical properties to the amorphous IrO_*x*_ and outperforms r-IrO_2_ which has been thermally treated at the same temperature, 500 °C, giving a clear indication that residual Li^+^ can prevent thermal deactivation of amorphous IrO_*x*_.

Cyclic voltammetry (CV) of IrO_*x*_ (Figure S8) showed characteristic Ir^3+^/Ir^4+^ and Ir^4+^/Ir^5+^ redox events at 0.9 and 1.2 V_RHE_ indicating facile redox processes at the surface and were also observed for Li-IrO_*x*_ at the same potentials.^25,43^ No characteristic features were observed for r-IrO_2_, suggesting that it is much less redox active and hence likely a poor catalyst for the OER.^44^ Catalyst stability was assessed through chronopotentiometry (10 mA cm^–2^) in Figure 4c; IrO_*x*_ and Li-IrO_*x*_ showed good stability over the 2 h test with degradation rates of 5.9 and 6.9 mV h^–1^, respectively. However, r-IrO_2_ showed complete deactivation within the first hour in agreement with previous reports.^18^ Longer term stability testing of IrO_*x*_ and Li-IrO_*x*_ at 10 mA cm^–2^ was conducted for 75 h (Figure 4d). During the first 25 h, IrO_*x*_ and Li-IrO_*x*_ showed similar performance with potentials ∼1.61 V_RHE_. The IrO_*x*_ catalyst then rapidly degraded after 25 h; in contrast, Li-IrO_*x*_ showed stable performance during at least 75 h with a degradation rate stabilizing at 0.3 mV h^–1^. Stability testing at higher current density (100 mA cm^–2^) also shows enhanced stability in Li-IrO_*x*_ with stable operation for 8 h when compared to only 4 h for IrO_*x*_ (Figure S9). Under high current density deactivation might not be necessarily related solely to catalyst corrosion but also to mechanical processes such as catalyst detachment induced by the high rate of bubble formation. Prolonged stability tests at high current densities are more reliable for freestanding or self-supported catalysts. Nevertheless, our catalytic results suggest that nanocrystalline iridate structures are comparative OER catalysts to iridium(oxy)hydroxides despite being exposed to temperatures which would normally deactivate amorphous systems. In this case, thermal treatment resulted in enhanced durability, which allows the desired balance between relatively high activity and long-term stability.

### Operando Spectroscopy

**Figure 5:**
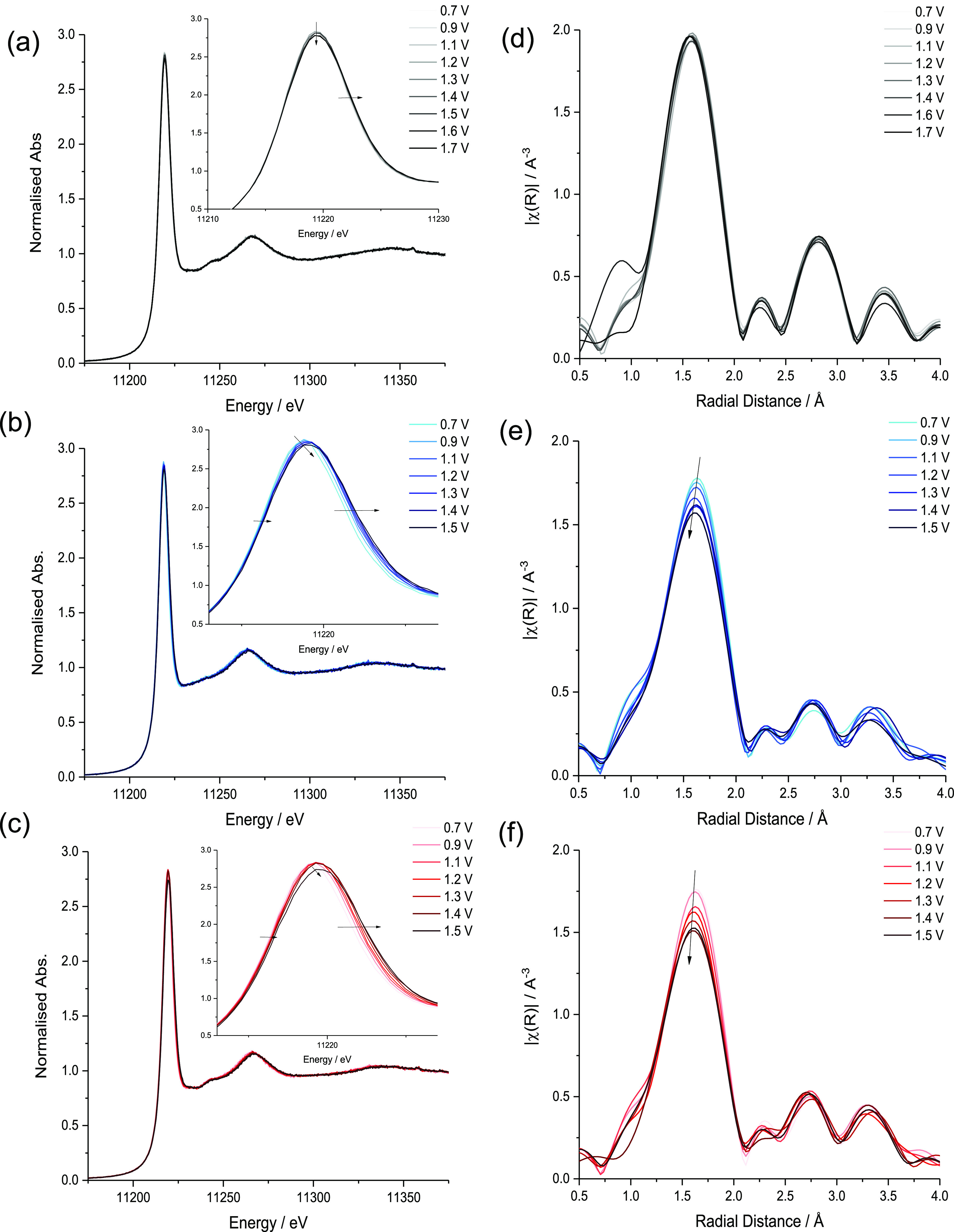
*In situ* normalized XANES spectra for (a) r-IrO_2_, (b) IrO_*x*_, and (c) Li-IrO_*x*_ recorded at different electrode potentials in 0.1 M HClO_4_. Insets show an expansion of the absorption peak to highlight the change in position. Fourier-transformed Ir EXAFS spectra of (d) r-IrO_2_, (e) IrO_*x*_ and (f) Li-IrO_*x*_ at given electrode potentials.

Czioska et al. recently conducted *operando* XAS of IrO_2_ and proposed that WL position is related to changes in surface speciation without modification of the IrO_2_ structure. Nattino et al. also reported WL shifts toward higher energies on deprotonation of surface hydroxyls, while WL height changes are caused by changes in d-band occupancy or emerging spectral features.^14 ,45^ O*perando* XAS spectra at 100 mV potential steps between 0.7 and 1.6 V were recorded for the three samples (Figure 5a–c). In contrast to Czisoka, we did not observe a WL height reduction and shift on applying “high” OER potentials (1.7 V_RHE_), proposed to result from increased Ir–Ir interactions through O-vacancy formation. Under applied potential, the WL of r-IrO_2_ remained largely unchanged, suggesting a minimal change in the Ir coordination. Due to reduced data quality, even simplified second shell structural models gave large errors meaning that structural information concerning the second coordination shell was limited.^40,46^ We applied a first shell Ir–O_6_ model by initially allowing CN, *R*, and 2σ^2^ of Ir–O to be floated and fixing the structural disorder factor at this value for subsequent fittings to avoid strong correlations between CN and structural disorder. Upon increasing potential from 0.9 to 1.7 V, Ir–O_1_ remained relatively consistent at 1.96–1.97 ± 0.01 Å as did the Ir–O coordination, 6.5 ± 0.4 at 0.9 V to 6.6 ± 0.8 at 1.7 V (Table S2) confirming the relative stability and lack of structural evolution.

Upon analysis of IrO_*x*_ with increasing potential (0.7–1.5 V) (Figure 5c,d), the WL position increased from 11,218.8 to 11,219.2 eV, suggesting more facile oxidation (deprotonation) or changes in surface Ir–O speciation. The FWHM increases from 7.3 to 8.1 eV giving asymmetrical broadening of the WL. The simplified fitting model (Table S3) suggests Ir–O_1_ of 2.02 ± 0.02 Å at 0.9 V and 2.00 ± 0.01 Å at 1.5 V with CN changing from 5.6 ± 1.1 at 0.9 V and 4.9 ± 0.5 at 1.5 V. Li-IrO_*x*_ (Figure 5e,f) shows the WL shift from 11,219.1 to 11,219.3 eV consistent with changes in surface speciation suggested by Czisoka et al.^45^ Above 1.2 V, the FWHM broadens significantly from 7.8 to 8.4 eV with the WL height decreasing from 2.85 at 1.2 V to 2.74 at 1.5 V, suggesting the development of a higher energy spectral feature and changes in the underlying structure. Similar to the IrO_*x*_ material, fitting suggests Ir–O_1_ of 2.02 ± 0.01 Å at 0.9 V and 2.00 ± 0.01 Å at 1.5 V with a significantly reduced Ir–O_1_ CN of 5.7 ± 1.1 at 0.9 V and 5.0 ± 0.6 at 1.5 V (Table S4). The fitting suggests that under the applied potential, IrO_*x*_ and Li-IrO_*x*_ behave similarly and form a significant number of O-vacancies. In the case of r-IrO_2_ and IrO_*x*_, these changes were recoverable on the cathodic sweeps (Figure S10), however, not fully for Li-IrO_*x*_ despite the stable OER activity. EXAFS fitting suggests changes in the Ir–O environment, which could be consistent with the exchange of Li^+^/H^+^ and protonation/deprotonation of both the surface and interplane Ir–O species.^25^

**Figure 6:**
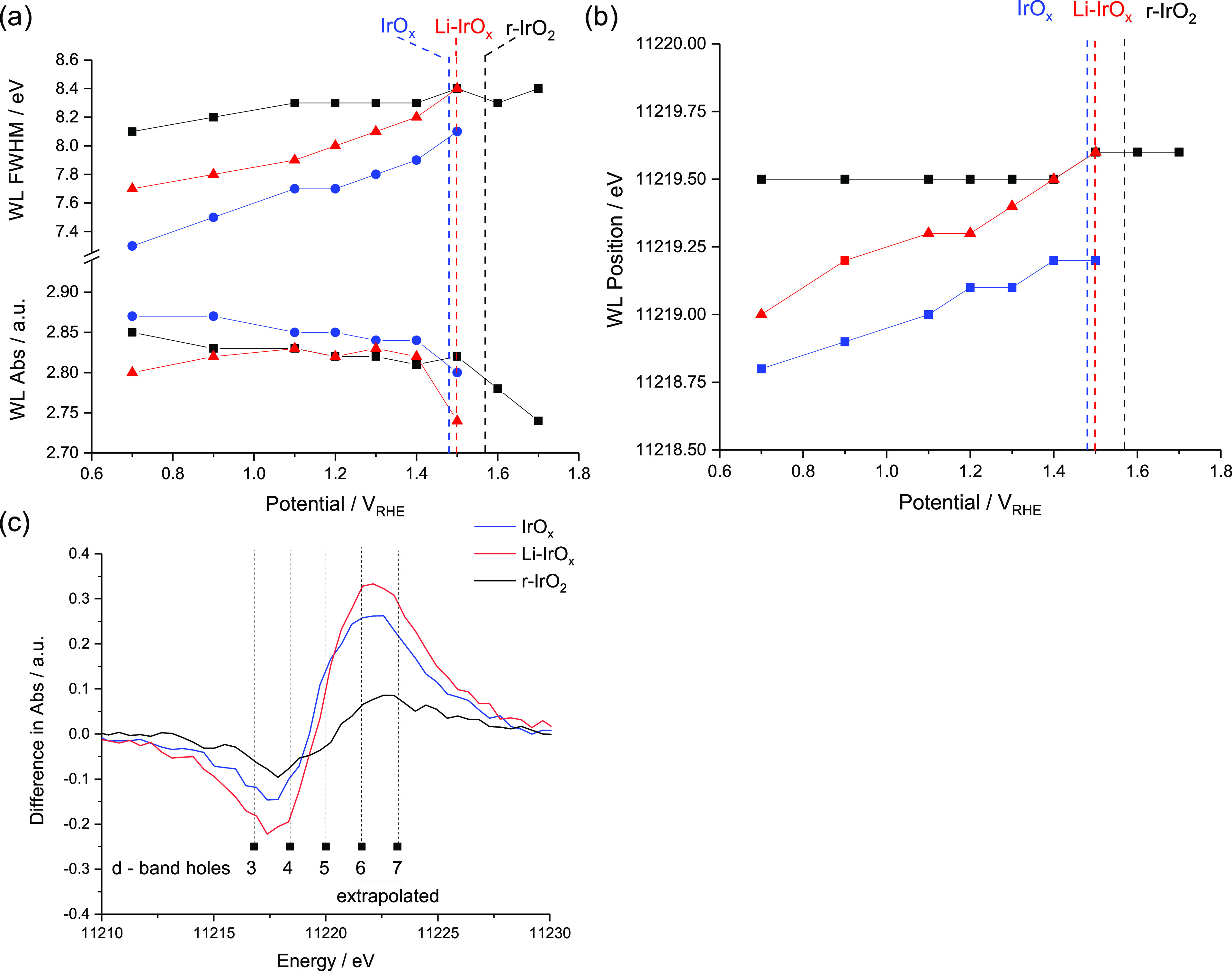
Numerical analysis of the *in situ* XANES spectra. (a) White line (WL) absorbance and FWHM, (b) WL position, and (c) difference in XANES spectra between the lowest and highest potentials studied.

Combining these observations, samples with higher binding energy Ir species measured by XPS (IrO_*x*_ and Li-IrO_*x*_) or oxygen species carrying an electron hole as determined by O K-edge XANES undergo WL shifts with increasing potential (Figure 6a,b), suggesting more redox active surfaces compared to r-IrO_2_. The highly active OER catalysts show WL asymmetric broadening to higher energy (by 0.3–0.9 eV) over the potential range which could suggest that FWHM broadening and decrease in WL intensity are not a result of the same processes as correlations between the WL position - intensity and FWHM - intensity were poor across all samples (*R*^2^ = <0.45) (Figure S11). This conclusion is also supported by the results of our ab initio simulations reported in the Supporting Information (Figure S12). A significant reduction in WL height is seen at the respective OER onset (dotted lines in I–V plots, Figure 6a)—this could represent a change in the d-band occupancy associated with O-vacancy formation without significant changes in Ir–Ir coordination. A good correlation (*R*^2^ = 0.93) is seen between the WL position and FWHM broadening meaning that deconvolution of a surface oxidation process (WL position) and evolution of higher energy spectral features (FWHM) is challenging. The shift and broadening could be a result of the development of oxidized Ir–O moieties consistent with many proposed OER mechanisms.

Difference spectra (Figure 6c) show asymmetry between the lowest and highest potential measurements. Superimposed are the WL positions derived from Figure S3. The r-IrO_2_ sample shows the smallest difference—implying that the Ir(IV) species measured by XPS and XAS are not redox active species in the bulk of the sample. IrO_*x*_ and Li-IrO_*x*_ show greater differences, suggesting that increased amounts of redox active Ir exist at the electrode/electrolyte interface. In the OER region, all samples have a significant increase in intensity at higher energies associated with asymmetric FWHM broadening, extrapolating beyond the standards recorded in Figure S3; it is clear that all samples show the formation of a similar feature or combination of features with an observed maximum intensity of ∼11,222.6 eV. We assign this to a more electron-deficient Ir–O species than the Ir(IV)–O in rutile-type IrO_2_ or the Ir(IV)–O–Li in Li-IrO_*x*_ or IrO_*x*_. This spectral transformation is consistent with the formation of metastable Ir(V)/μ_1_-oxyl species proposed as OER intermediate species.^28^ High OER activity and more pronounced structural changes for IrO_*x*_ and Li-IrO_*x*_ correlate well with the enhanced redox dynamics observed by CV. A more reactive surface and higher OER activity can be correlated to the initial Ir speciation in these systems, with the higher binding energy component more susceptible to undergo redox dynamics leading to the evolution of O_2_.^47−51^ We can conclude that all three materials undergo similar evolution under polarization but to different degrees with an OER mechanism involving the formation of Ir(V)/μ_1_-oxyl species as well as the generation of oxygen vacancies (shift of the L_3_-edge to a higher energy in the X-ray absorption near-edge structure (XANES) spectrum and decrease of its intensity in the experimental XANES data and in the simulated XANES in Figure S10). Similar to previous work, we postulate that these Ir(V)/μ_1_-oxyl species are formed under anodic potentials via deprotonation and oxidation of the surface Ir–OH of r-IrO_2_ or Ir–O–Li species on Li-IrO_*x*_ and IrO_*x*_. Due to their electrophilic character, Ir(V)/μ_1_-oxyl species are susceptible to undergo nucleophilic attack by neighboring OH species forming the intermediate O–O bond that results in the evolution of oxygen and formation of the vacancies. We suggest that the exceptional stability of the layered Li-IrO_*x*_ system might be explained by the fact that this transformation becomes anisotropic for a layered structure, which retains a stable backbone structure of Ir-oxo layers, whereas oxyl species are formed from the longer Ir–O bonds seen in these systems in the O K-edge NEXAFS spectra in Figure 1d. The presence of longer Ir–O bonds, possibly due to the polarizing effect of Li^+^ species, could lead to these being preferentially involved in the O–O bond formation. It is possible that these oxygen ligands are oriented perpendicularly to the layers and located within the interlayer space where the oxygen turn over preferentially takes place. In contrast, in the case of IrO_*x*_, this transformation involves both axial and equatorial oxygen species, ultimately leading to a disruption of the structure and dissolution of Ir species upon water ligation causing a deterioration of the performance.

### Thermal Processing of Li-IrO_*x*_

**Figure 7:**
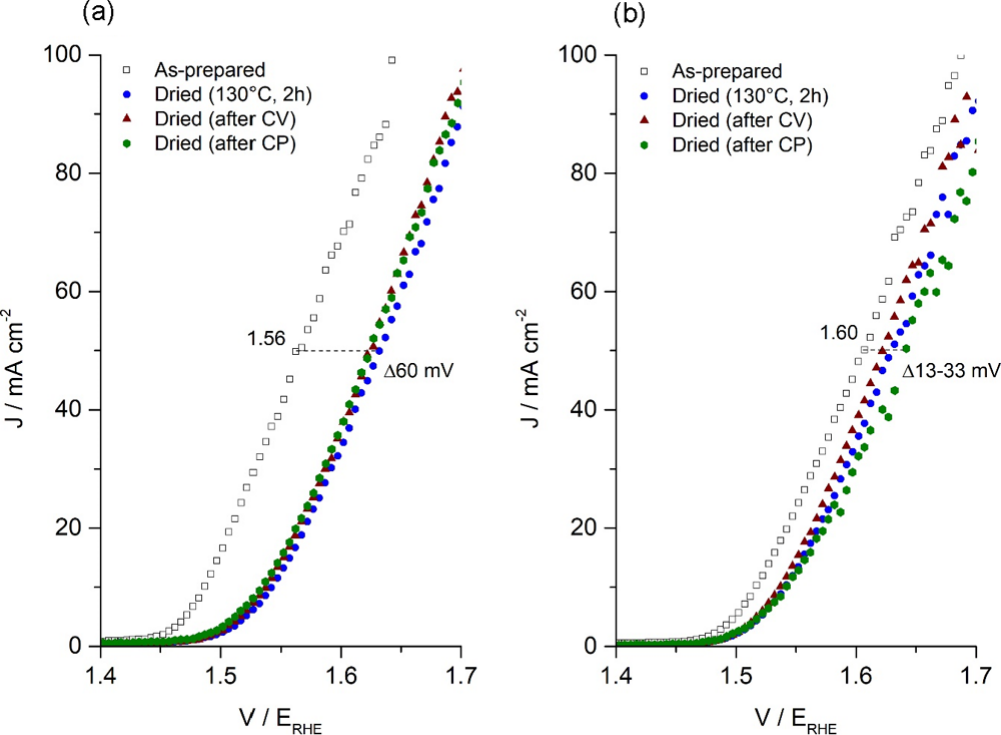
Effect of electrode drying (130 °C, 2 h) on the OER activity (LSV, 1.2–1.7 V_RHE_, 5 mV s^–1^) compared to as-prepared electrodes for (a) IrO_*x*_ and (b) Li-IrO_*x*_.

Li-IrO_*x*_ which has been thermally treated at 500 °C retains high activity comparable to IrO_*x*_ which is dried at room temperature to prevent conversion to rutile. Due to the sensitivity toward drying temperatures, extreme care must be taken in the preparation of electrodes, which becomes challenging at scale when dry weight is used to estimate Ir loadings requiring elevated drying temperatures (>100 °C).^52,53^Figure 7a records the LSV of IrO_*x*_ electrodes dried at room temperature and 130 °C for 2 h. The results suggest a deactivation of IrO_*x*_ with increased drying temperature with a change of 60 mV needed to achieve 50 mA cm^–2^. In contrast, Li-IrO_*x*_ which has been thermally treated at 500 °C shows a change of just 13 mV at comparable current densities suggesting a high stability to harsher electrode preparation conditions (Figure 7b).

## Conclusions

Many studies utilize alkali metals as precipitating agents for the synthesis of iridium oxides used in electrocatalytic reactions. This study shows for the first time that the residual Li^+^ can supress the formation of rutile IrO_2_ on the thermal treatment of amorphous oxides and hence deactivation for the OER and instead direct the transformation to produce a nanocrystalline layered Li-iridate at relatively mild conditions. This iridate shows comparable activity to amorphous iridium oxyhydroxides despite being treated at 500 °C and enhanced stability over extended reaction times. The dynamics of the L_3_-edge spectroscopic features of this electrocatalyst under the oxygen evolution reaction are like those observed for the rutile IrO_2_ and the amorphous IrO_*x*_ indicating similar structural dynamics and thus reaction mechanism. The incorporation of structural alkali metal dopants into layered oxides provides a route to not only stabilize high activity materials but also to controllably tune the electronic properties of metal oxide surfaces. We show herein that a layered structure allowed us to realize orthogonality between activity and stability, the latter being a major bottleneck in the design of OER active electrocatalysts.

## Experimental Section

### Synthesis of Ir-Based Materials

Amorphous iridium oxyhydroxide (IrO_*x*_) was prepared following a precipitation method reported previously.^18^ 299 mg of IrCl_3_ hydrate (1 mmol) and 591 mg of Li_2_CO_3_ (8 mmol) were dissolved in 10 mL of deionized water and stirred for 16 h at 25 °C. A further 10 mL of deionized water was added, followed by heating to reflux for 3 h. The mixture was allowed to cool to room temperature. The formed precipitate was recovered by filtration and washed with 1 L of cold water and 1 L of hot water. The solid was dried in an open vessel at 25 °C for 16 h. Rutile IrO_2_ (r-IrO_2_) was prepared by annealing IrO_*x*_ in static air (500 °C, 10 °C min^–1^, 3 h) . Lithium-doped IrO_*x*_ (Li-IrO_*x*_) was synthesized as follows: 299 mg of IrCl_3_ hydrate (1 mmol) and 591 mg of Li_2_CO_3_ (8 mmol) were dissolved in 10 mL of deionized water and stirred for 16 h at 25 °C. A further 10 mL of deionized water was added, followed by heating to reflux for 3 h. After cooling to room temperature, the precipitate was recovered by filtration without any washing step, dried in an open vessel (25 °C, 16 h), and annealed in static air (500 °C, 10 °C min^–1^, 3 h). Excess Li_2_CO_3_ and Cl contamination were removed by washing with 1 L of hot and 1 L of cold deionized water. Finally, the material was dried in an open vessel at 25 °C for 16 h.

### Catalyst Characterization

Powder XRD was performed on a (θ–θ) PANalyticalX’pert Pro powder diffractometer fitted with a position-sensitive detector using a Cu Kα radiation source (40 keV, 40 mA). An *in situ* Anton Parr XRK900 cell was used to monitor the crystallization while heating to 575 °C under static air. At each temperature step, the heating ramp (10 °C min^–1^) was paused and the sample was held at that temperature, while a diffraction pattern was measured between 15 and 80° 2θ.

Samples were deposited dry on the TEM grids and characterized in an aberration-corrected Titan 80–300 operated at 200 kV.

Ir 4f XPS and O K-edge measurements were performed on the pelletized samples at the AP-XPS end station of the ISISS beamline (BESSY II) at 10^–7^ mbar base pressure. Ir 4f spectra were recorded by collecting photoemitted electron with a kinetic energy of 200 eV at a pass energy of the electron analyzer set to 20 eV. The energy of the spectra was calibrated against the Fermi level measured at the same excitation energy. The spectra were fitted using the fitting developed in refs (6, 7, 53, 10) after Shirley background subtraction. The O K-edge NEXAFS spectra were collected in total electron yield mode by detecting the sample drain current with a current amplifier. The relative energy calibration of the spectra was checked using features in the drain current of the last refocusing mirror of the beamline, then setting the pre-edge feature of the rutile type sample to 530 eV, and shifting the other spectra for the same amount. Spectra were normalized to the maximum intensity at 546 eV, after linear background subtraction.

XAS spectra were recorded in fluorescence mode at the Ir L_3_ edge, at the B18 beamline of the Diamond Light Source, at Harwell, U.K. Prior to ex situ and *in situ* XAS analysis, 56 μL of catalyst ink dispersion (5 mg catalyst in 1.23 mL of water, 1.23 mL of ethanol, and 40 μL of Nafion solution) was drop-cast on a 5 mm × 5 mm square of carbon cloth (Sigracet 39 AA, 50 g m^–2^, Fuel Cell Store) to obtain a catalyst loading of 150 mg_cat_ cm^–2^. Five scans were recorded, and the averaged signal was used for data analysis. The measurements were performed using a QEXAFS setup with a fast-scanning Si (111) double crystal monochromator. For the *in situ* measurements, the time resolution of the data acquisition was ∼60 s/spectrum. XANES and EXAFS data were interpreted using IFEFFIT with Demeter software package (Athena and Artemis) using structural models available from ICDD as cited in the text. *In situ* XAS measurements were carried out using a sealed cell described previously in literature filled with a 0.1 M HClO_4_ electrolyte. A Pt wire and a Ag/AgCl electrode were used as counter and reference electrodes, respectively.^54^

The density functional theory (DFT) calculations presented in Figure S11 were performed using the plane-wave pseudopotential DFT method available within the code CASTEP. Generalized-gradient approximation for the exchange-correlation energy was selected in the form of the solid-state revised PBE functional. Norm-conserving pseudopotentials used for PBE calculations were generated self consistently. A kinetic energy cutoff of 1150 eV for the wave function and a (10 × 10 × 10) Monkhorst-Pack *k*-point grid were determined as parameters for converged calculations. After introducing the localized structural defect (either vacancy of additional, bound oxygen), the structures have been relaxed to a tolerance energy of 1 × 10^–6^ eV/atom and forces of 2 × 10^–2^ eV/atom. A slightly extended k-point grid (12 × 12 × 12) was used for the simulation of spectroscopy results. XANES spectra were computed by extracting the matrix elements for electronic inter-band transitions from the ground-state DFT perturbed with the inclusion of the local effects of 2p core-hole, as available in the code. A transition broadening, because of instrumental resolution (Gaussian) and core-lifetime (Lorentzian) effects, was applied with values of 0.2 and 5 eV FWHM, respectively.

### Catalytic Activity toward the Oxygen Evolution Reaction

Catalysts were tested on a three-electrode setup in a 0.1 M HClO_4_ electrolyte. A coiled Pt wire (127 μm diameter, 99.99%, Sigma-Aldrich) was used as the counter electrode, a glassy carbon electrode with PEEK isolation (10 mm OD, 5 mm ID, IJ Cambria Scientific Ltd.) was used as the working electrode, and a calomel electrode ([Cl^–^/Hg_2_Cl_2_/Hg/Pt], IJ Cambria Scientific Ltd., model CHI-150) was used as the reference electrode. To prepare the catalyst ink, 5 mg of catalyst, 1.23 mL of water, 1.23 mL of ethanol, and 40 μL of Nafion solution were sonicated for 30 min to ensure a complete dispersion. 10 μL of the catalyst ink was drop-cast onto the working electrode and dried in the open at 25 °C for 16 h. The catalyst activity toward OER was measured by linear sweep voltammetry (LSV, 1.2 to 1.7 V_RHE_ at 5 mV s^–1^). Catalyst stability was assessed by CV (0.7 to 1.7 V_RHE_, 50 mV s^–1^, 50 cycles) and chronopotentiometry (CP, 2 h at 10 mA cm^–2^). Stability testing on the longer timescale was conducted by chronopotentiometry at 10 mA cm^–2^ for 75 h using an H-cell setup. The double-layer capacitance (*C*_DL_) was obtained from CVs in the 0.4–0.5 V_RHE_ region at different scan rates (2, 5, 10, 20, 40, and 80 mV s^–1^). The ECSA was obtained by dividing the *C*_DL_ by the specific capacitance in acid media (*C*_s,ac_ = 0.035 mF cm^–2^). Tafel slopes were derived from semi-steady-state conditions obtained from LSV measurements performed at low overpotential (1.43–1.56 V_RHE_, 0.5 mV s^–1^). Reported values are expressed against the reversible hydrogen electrode (RHE).

## Supporting Information Available

XRD, electron microscopy, X-ray absorption, EXAFS fittings, and electrochemistry (PDF)
