# Supplementary material for: Lithium-Directed Transformation of Amorphous Iridium (Oxy)hydroxides To Produce Active Water Oxidation Catalysts
Source: J Am Chem Soc. 2023 Mar 9;145(11):6398–409. doi: 10.1021/jacs.2c13567 (PMC10037335; doi:10.1021/jacs.2c13567)

Supplementary Information

Lithium Directed Transformation of Amorphous Iridium (oxy)hydroxides to Produce Active Water Oxidation Catalysts.

Jonathan Ruiz Esquius[a,b], David J. Morgan[a], Gerardo Algara Siller[c#], Diego Gianolio[d], Matteo Aramini[d], Leopold Lahn[e,f] Olga Kasian[e,f], Simon A. Kondrat[g], Robert Schlögl[c,h], Graham J. Hutchings[a], Rosa Arrigo[i] and Simon J. Freakley[j]*

[a] *Max Planck-Cardiff Centre on the Fundamentals of Heterogeneous Catalysis FUNCAT, Cardiff Catalysis Institute, School of Chemistry, Cardiff University, Main Building, Park Place, Cardiff, CF10 3AT, UK..*

[b] *International Iberian Nanotechnology Laboratory, Av. Mestre José Veiga, Braga 4715-330, Portugal.*

[c] *Department of Inorganic Chemistry, Fritz Haber-Institut der Max-Planck-Gesellschaft, 14195 Berlin, Germany*

[d] *Diamond Light Source Ltd, Harwell Science and Innovation Campus, Fermi Ave, Didcot, OX11 0DE, UK*

[e] *Helmholtz-Zentrum Berlin GmbH, Helmholtz Institut Erlangen-Nürnberg, Cauerstr. 1, 91058 Erlangen, Germany*

[f] *Department of Materials Science and Engineering, Friedrich-Alexander-Universität Erlangen-Nürnberg, 91058 Erlangen, Germany*

[g] *Department of Chemistry, Loughborough University, Epinal Way, Loughborough, Leicestershire, LE11 3TU, UK*

[h] *Department of Heterogeneous Reactions, Max Planck Institute for Chemical Energy Conversion, 45470, Mulheim an der Ruhr, Germany*

[i.] *School of Science, Engineering and Environment, University of Salford, M5 4WT Manchester, U.K*

[j] *Department of Chemistry, University of Bath, Claverton Down, Bath, BA2 2AY, UK.*

# Now at AG Strukturforschung / Elektronenmikroskopie. Institut für Physik. Humboldt-Universität zu Berlin. Newtonstraße 15 12489 Berlin, Germany

Corresponding author – s.freakley@bath.ac.uk

Figure S1 - XRD of commercially available rutile IrO_2 (Sigma Aldrich) and synthesised r-IrO_2.

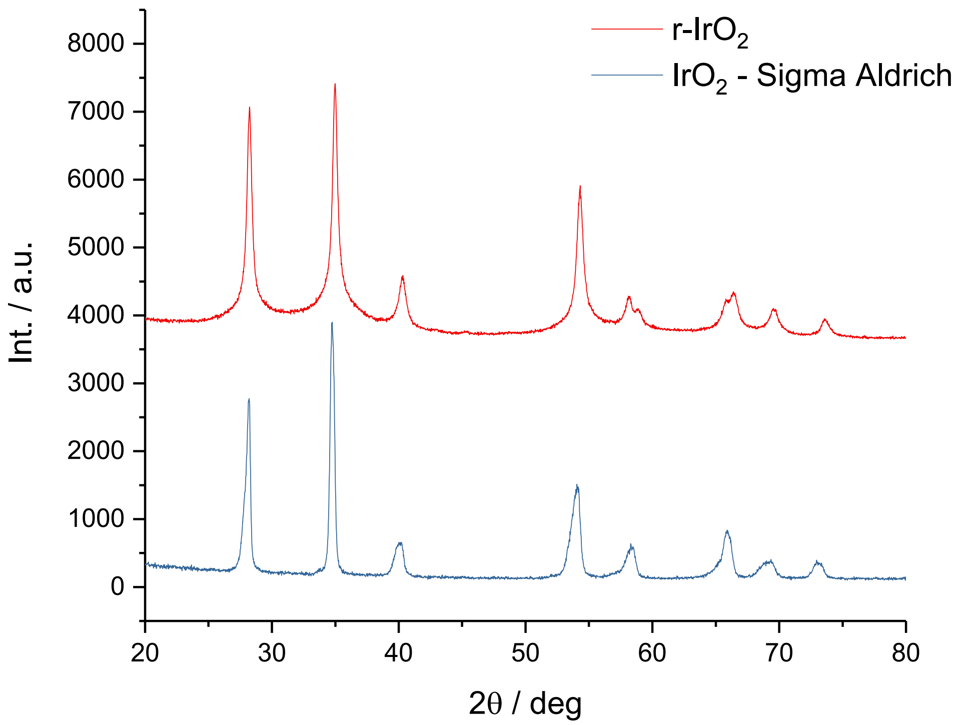

Figure S2 - Electron microscopy characterisation of fresh IrO_x. High resolution transmission electron microscopy image, electron diffraction pattern and its radial profile show the amorphous character of the sample before heating. Scale bar 10 nm.

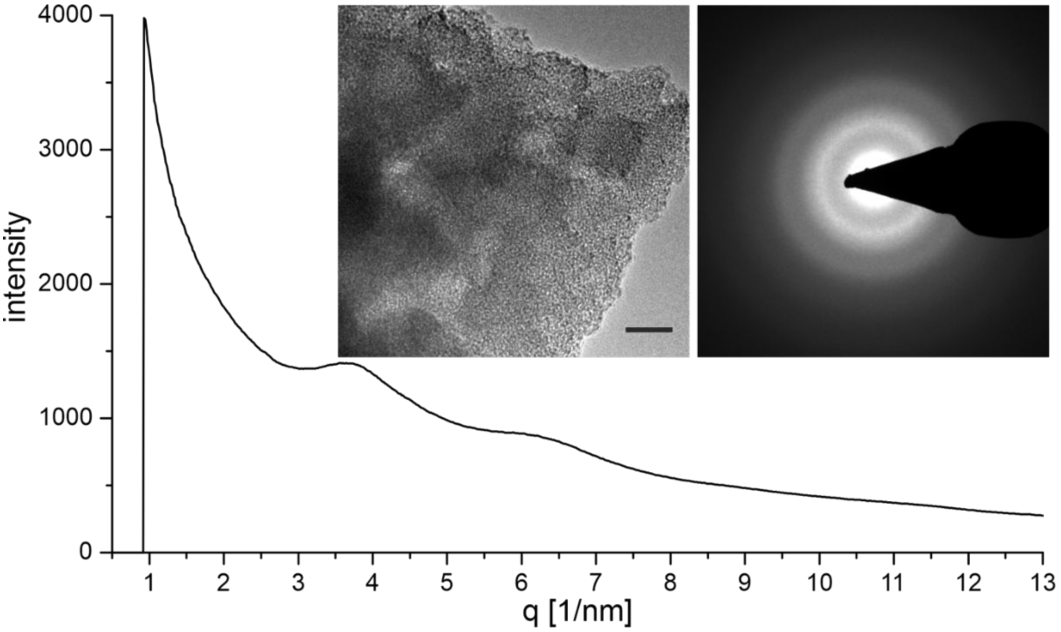

Figure S3 - Correlation between white line position (determined by the maximum in the second derivative) and edge position (determined by the maximum in the first derivative) as a function of the formal d-band hole count compared to metallic iridium ($5d^7$), $IrCl_3$ ($5d^6$) and IrO_2 ($5d^5$) standards.

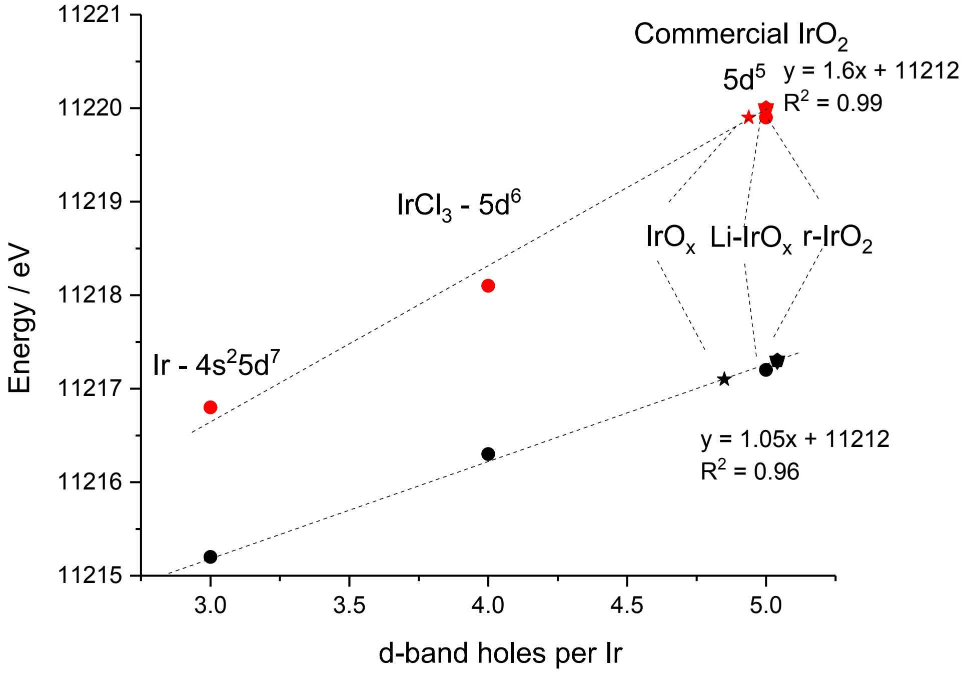

Table S1- EXAFS model fitting parameters for fresh r-IrO_2, IrO_x and Li-IrO_x and $IrCl_3$ from data measured at the Ir-L_3 edge.

Sample	Scattering Path	CN	R(Å)	$2\sigma^2$ ($Å^2$)	S_o^2	E_f (eV)	R_{factor} + (χ^2)
$IrCl_3$	Ir-Cl_1	6*	2.33(1)	0.003(1)	0.79	7.5(3)	0.018
r-IrO_2	Ir-O	6.1(1.6)	1.97(2)	0.002(10	0.79*	11.8(6)	0.04 (2830)
	Ir-Ir_1	3.0(1.5)	3.14(2)	0.004(2)			
	Ir-Ir_2	6.8(1.2)	3.55(1)	0.003(1)			
IrO_x	Ir-O	5.9(4)	2.013(4)	0.005(1)	0.79*	12.5(5)	0.010 (2961)
	Ir-Ir	6*	3.14(2)	0.013(2)			
	Ir-O_2	6*	3.64(2)	0.007 (2)			
IrO_x +Li path	Ir-O	6.1(4)	2.017(5)	0.005(1)		13.2(7)	0.008 (2810)
	Ir-Ir	6*	3.13(2)	0.014(2)			
	Ir-O_2	6*	3.64(2)	0.004(3)			
	Ir-Li	6*	2.83(6)	0.025(20)			
Li-IrO_x	Ir-O	6.3(5)	2.000(6)	0.005(1)	0.79*	12(1)	0.028 (2128)
	Ir-Ir_1	6*	3.09(2)	0.011(2)			
	Ir-O_2	6*	3.62(3)	0.006(3)			
Li-IrO_x + Li paths	Ir-O	6.3(5)	2.000(6)	0.006(1)		11.2(7)	0.014 (1438)
	Ir-Ir_1	6*	3.07(1)	0.009(1)			
	Ir-O_2	6*	3.66(3)	0.009(4)			
	Ir-Li_1	6*	2.90(2)	0.003(2)			
	Ir-Li_2	6*	3.35(6)	$0.005^{\#}$			

*Ir-Cl CN fixed at 6 to determine S_o^2 at a value of 0.79. # $2\sigma^2$ determined from value of Ir-Li_1 x 1.4.

Figure S4 – Fourier transformed fits of EXAFS and k-space for a) r-IrO_2, b) IrO_x and c) Li-IrO_x based on a two-shell model including Ir-O, Ir-Ir and Ir-Li paths between R = 1.5 – 3.5 A, k 3 – 14. d) Plots of the Imaginary part of the Fourier Transformed EXAFS data with individual contributions of fitted scattering paths. (Left) IrOx fitted including single Li path. (right) LiIrOx fitted including two Li paths within layered structure

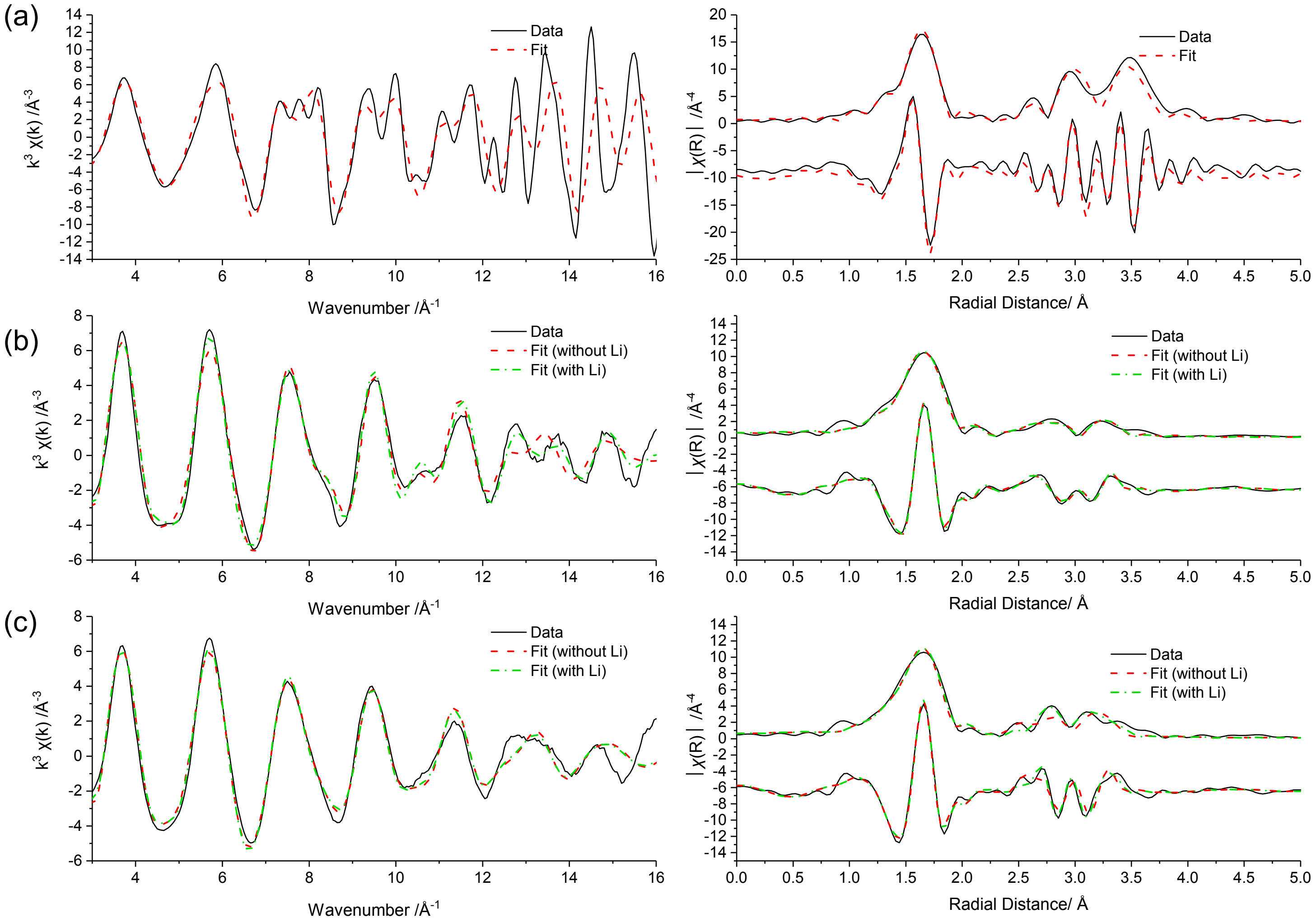

Figure S5 - Plots of the Imaginary part of the Fourier Transformed EXAFS data with individual contributions of fitted scattering paths. (Left) IrOx fitted including single Li path. (right) LiIrOx fitted including two Li paths within layered structure

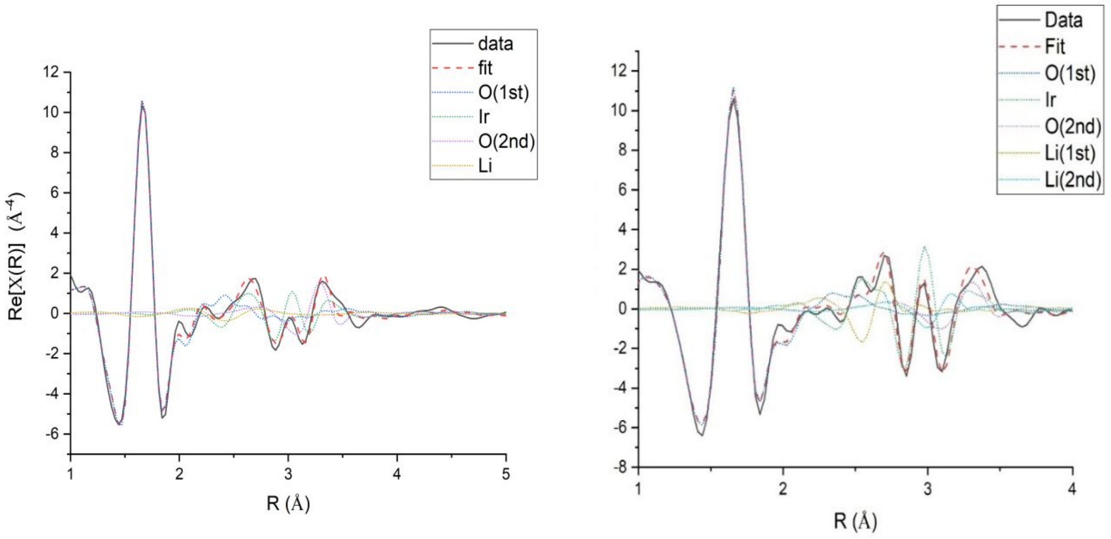

Figure S6– Ball and stick representations of a) rutile IrO_2 [ICSD 56009] and b) β-Li_2IrO_3 [ICSD 246025] used to model the structure of the synthesised Li-IrO_x.

(a)

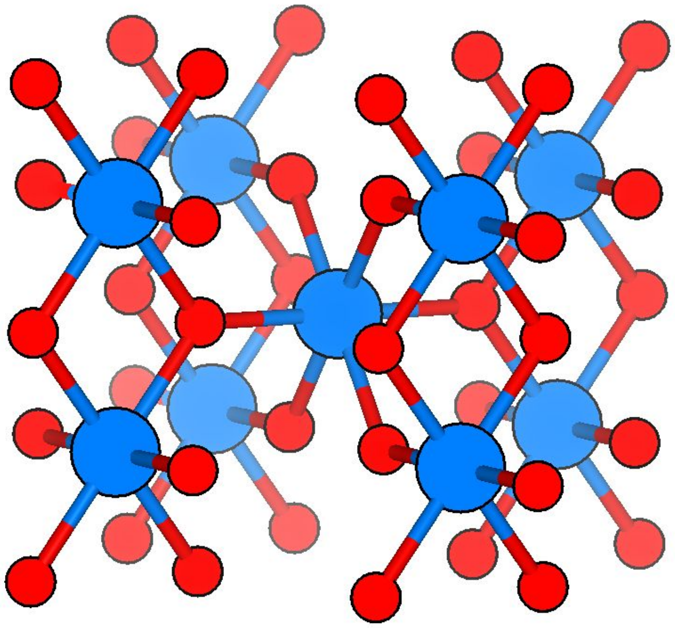

(b)

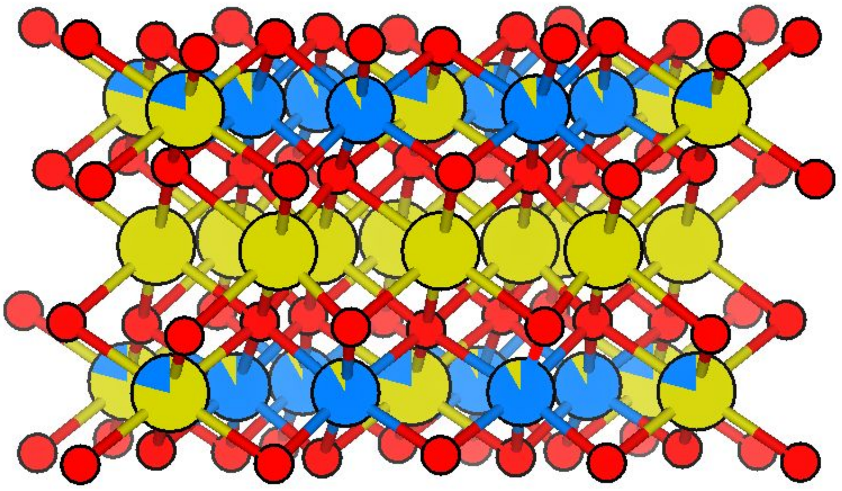

Figure S7 – Process for the determination of the double layer capacitance (C_{DL}) for IrO_x and Li-IrO_x catalysts through cyclic voltammetry (CV) from a) CV curves (0.3-1.4 V_{RHE}, 50 mV s^{-1}) to determine a region free of Faradaic processes. CV curves recorded between 0.4-0.5 V_{RHE} at different scan rates (2-80 mV s^{-1}) for b) IrO_x and c) Li-IrO_x and d) double layer capacitance obtained from plotting the scan rate against the average between anodic and cathodic current at 0.45 V_{RHE}.

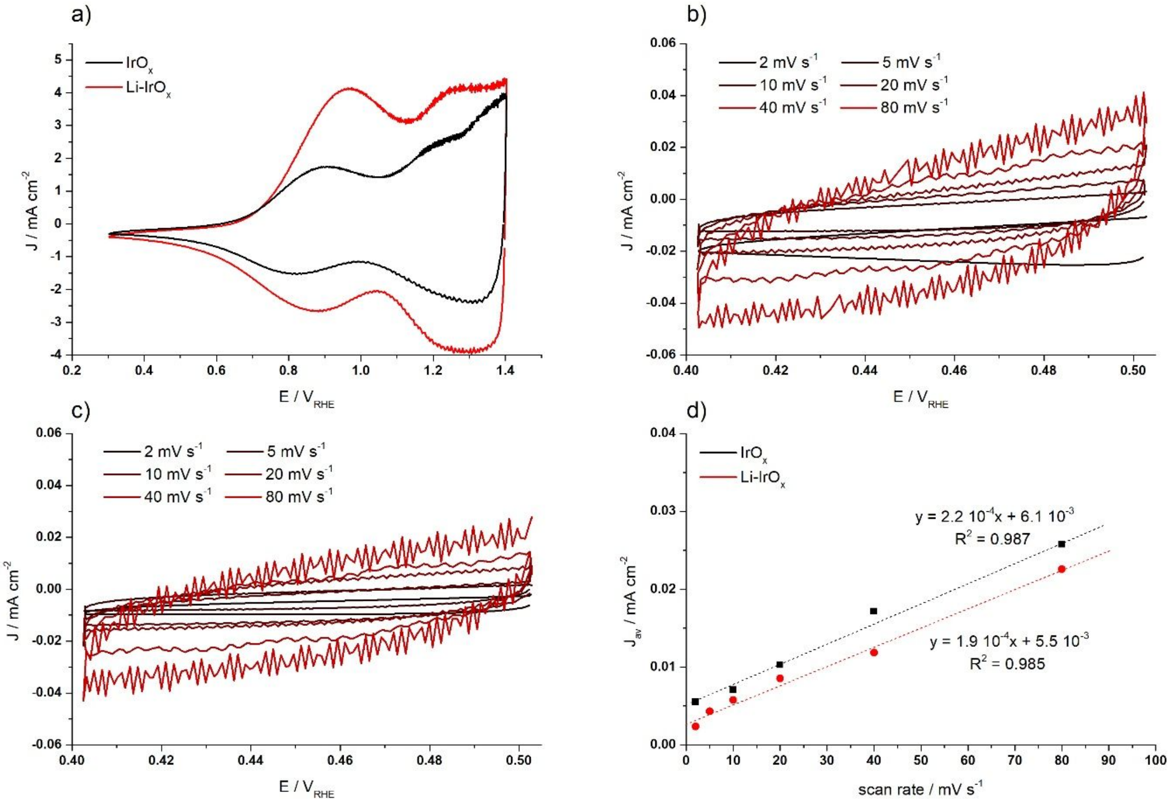

Figure S8 – CV curves recorded over consecutive cycles (50 mV s^{-1}, 0.3-1.4 V_{RHE}, 50 cycles) for a) IrO_x b) Li-IrO_x and c) r-IrO_2.

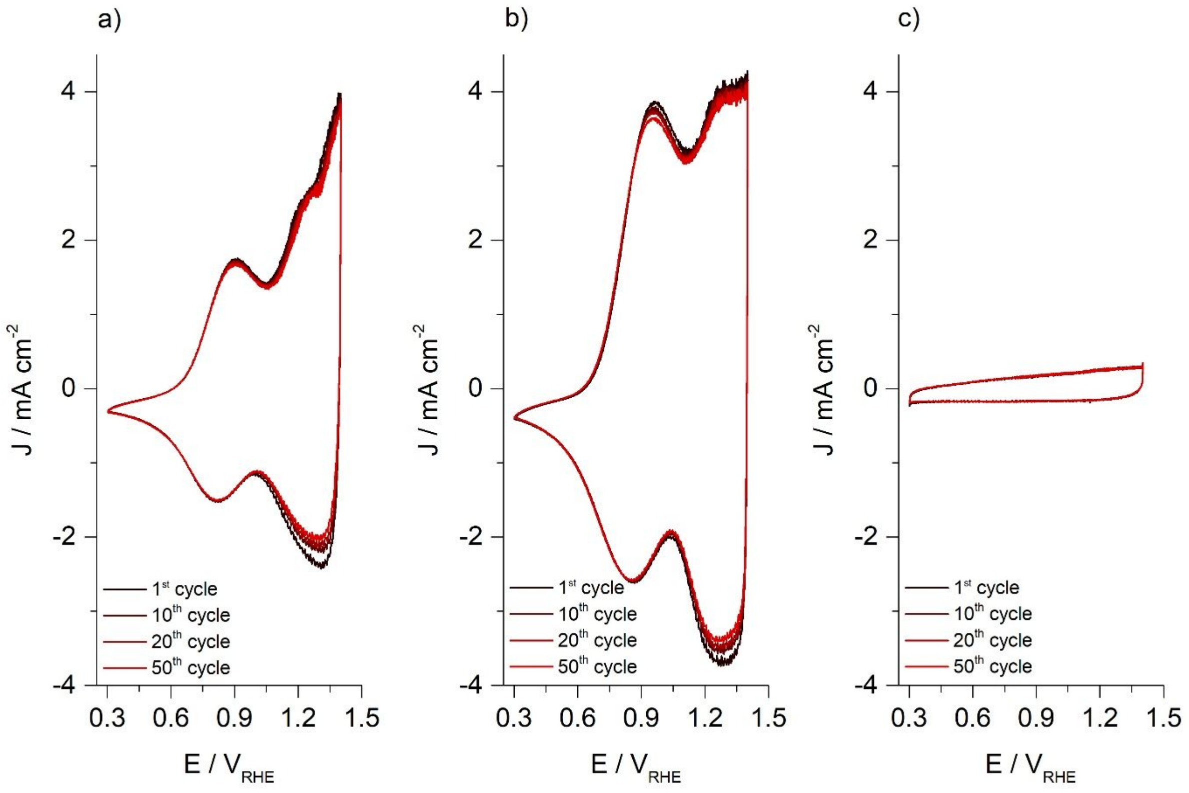

Figure S9 – Chronopotentiometry at 100 mA cm^{-2} towards OER in 0.1 M $HClO_4$ electrolyte

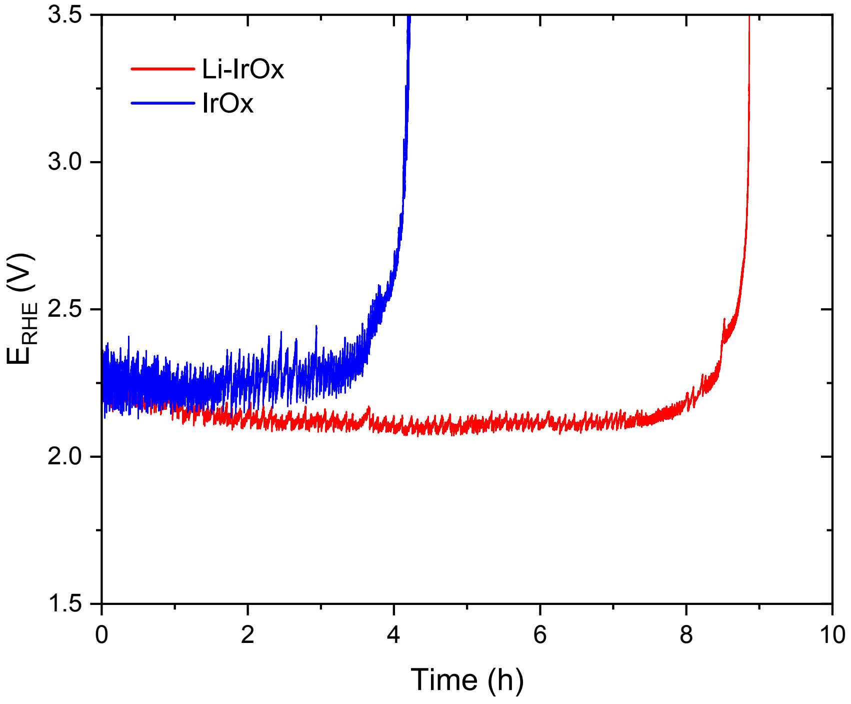

Table S2 – EXAFS model fitting parameters for first shell Ir-O of IrO_2 measured *in situ* at the Ir-L_3 based on IrO_2 [ICSD 56009] and with floated CN and R. Fitted data range k 3-12 and R 1.5 – 2.5 Å.

**Potential / V_{RHE}**	**Scattering Path**	**CN**	**R(Å)**	**2σ² (Å²)**	**S_o^2**	**E_f (eV)**	**R_{factor}**	**χ^2**
0.9	Ir-O	6.5 ± 0.4	1.97 ± 0.01	0.003 ± 0.002		9.4 ± 1.3	0.007	95
1.0	Ir-O	6.6 ± 0.4	1.97 ± 0.01			8.4 ± 1.7	0.009	57
1.1	Ir-O	6.4 ± 0.5	1.98 ± 0.01			10.1 ± 1.9	0.017	67
1.2	Ir-O	6.6 ± 0.5	1.97 ± 0.01			9.3 ± 1.5	0.011	62
1.3	Ir-O	6.5 ± 0.5	1.97 ± 0.01	0.003*	0.79*	9.6 ± 1.8	0.016	50
1.4	Ir-O	6.5 ± 0.5	1.97 ± 0.01			9.4 ± 1.6	0.012	37
1.5	Ir-O	6.6 ± 0.7	1.97 ± 0.02			9.5 ± 2.3	0.023	128
1.6	Ir-O	6.2 ± 0.6	1.98 ± 0.02			10.6 ± 0.6	0.026	101
1.7	Ir-O	6.6 ± 0.8	1.96 ± 0.01			9.6 ± 2.7	0.032	203

* fixed variable

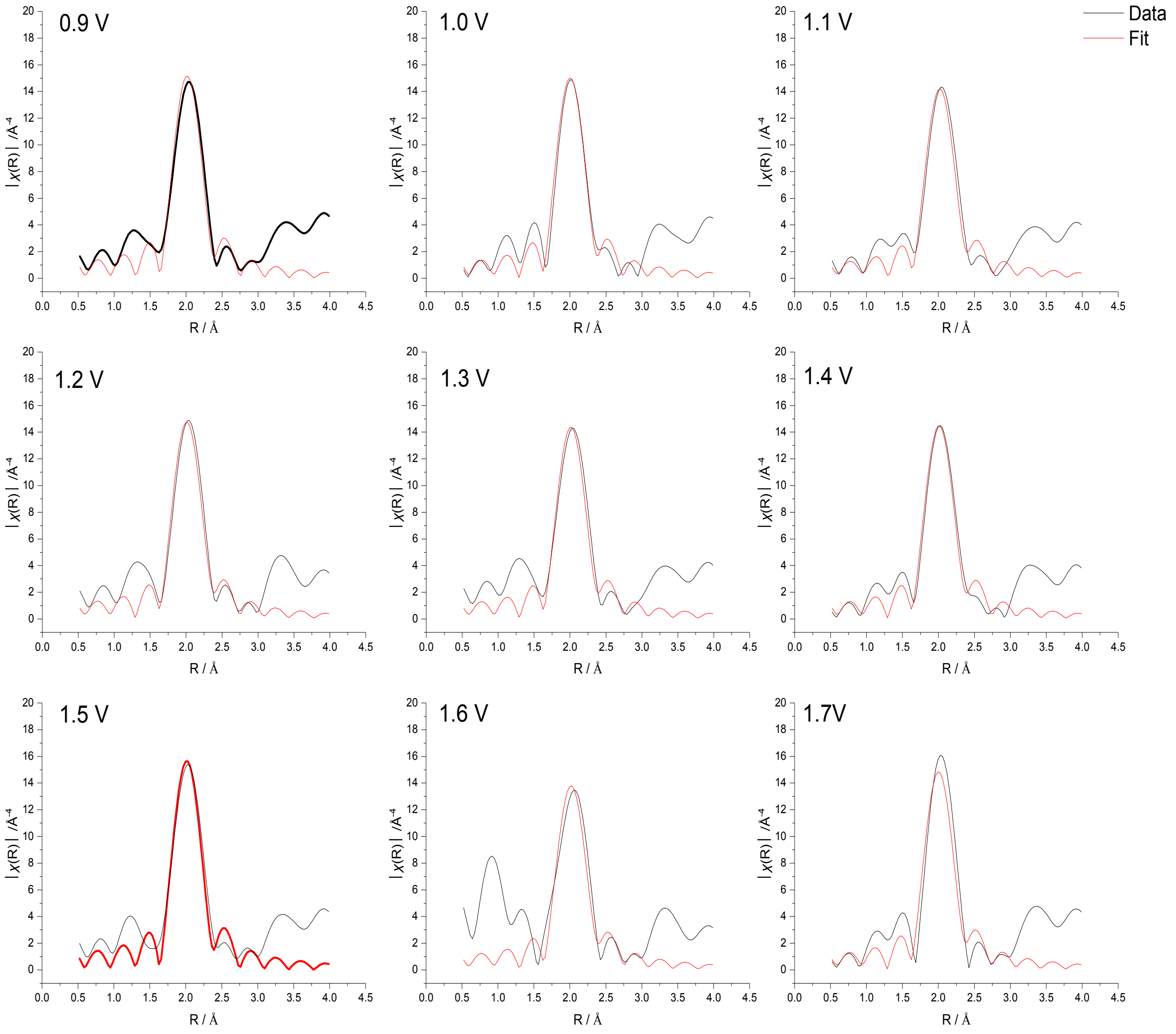

Table S3 – EXAFS model fitting parameters for of IrO_x measured *in situ* at the Ir-L_3 edge based on IrO_2 [ICSD 56009] and with floated CN and R. Fitted data range k 3-12 and R 1.5 – 2.5 Å.

Potential / V_{RHE}	Scattering Path	CN	R(Å)	$2\sigma^2$ (Å^2)	S_o^2	E_f (eV)	R_{factor}	χ^2
0.9	Ir-O	5.6 ± 1.1	2.02 ± 0.02	0.002 ± 0.001		11.7 ± 2.1	0.015	81
1.1	Ir-O	5.5 ± 0.4	2.02 ± 0.01			11.9 ± 1.4	0.010	33
1.2	Ir-O	5.2 ± 0.4	2.01 ± 0.01		0.79*	11.3 ± 1.6	0.014	46
1.3	Ir-O	5.2 ± 0.4	2.02 ± 0.01	0.002*		12.3 ± 1.8	0.021	39
1.4	Ir-O	5.0 ± 0.5	2.00 ± 0.01			13.1 ± 2.1	0.030	90
1.5	Ir-O	4.9 ± 0.5	2.00 ± 0.01			12.7 ± 2.2	0.026	31

* fixed variable

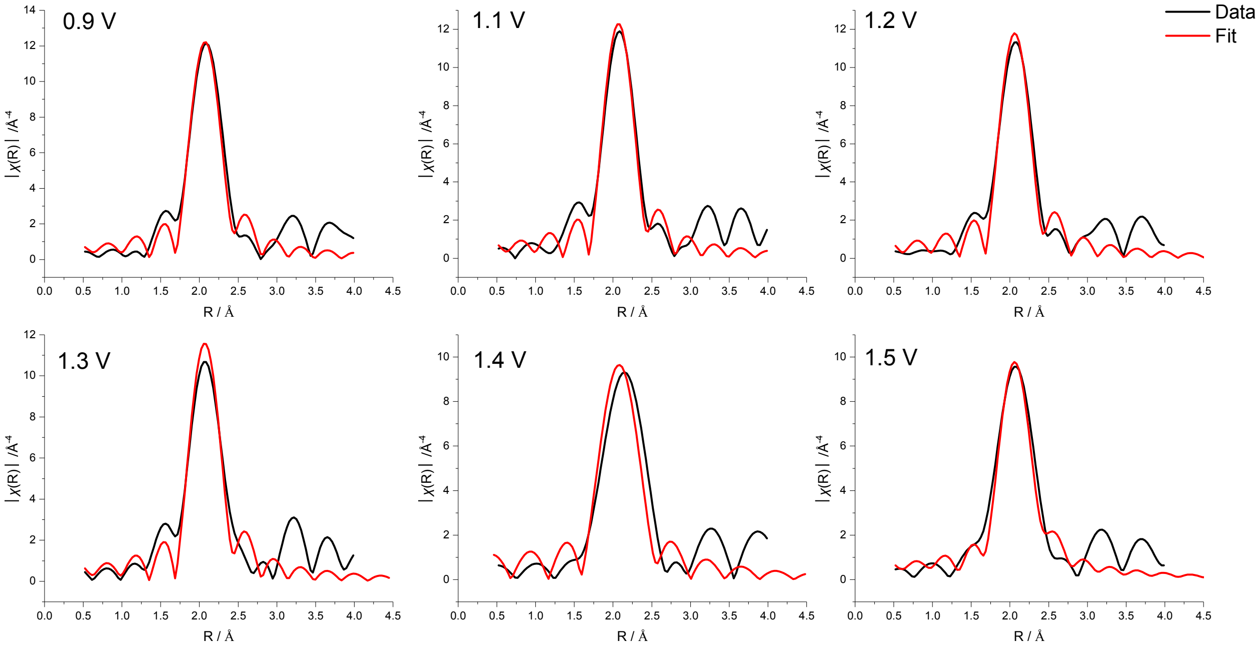

Table S4 – EXAFS model fitting parameters for of Li-IrO_x measured *in situ* at the Ir-L_3 edge based on IrO_2 [ICSD 56009] and with floated CN and R. Fitted data range k 3-12 and R 1.5 – 2.5 Å.

**Potential / V_{RHE}**	**Scattering Path**	**CN**	**R(Å)**	**$2\sigma^2$ (Å^2)**	**S_o^2**	**E_f (eV)**	**R_{factor}**	χ^2
0.9	Ir-O	5.7 ± 1.1	2.02 ± 0.02	0.003 ± 0.001		12.1 ± 2.3	0.015	64
1.1	Ir-O	5.4 ± 0.4	2.02 ± 0.01			12.7 ± 1.7	0.015	45
1.2	Ir-O	5.4 ± 0.4	2.01 ± 0.01		0.79*	11.9 ± 1.7	0.013	37
1.3	Ir-O	5.2 ± 0.4	2.01 ± 0.01	0.003*		12.5 ± 1.8	0.017	49
1.4	Ir-O	5.0 ± 0.5	2.01 ± 0.01			13.0 ± 1.9	0.019	46
1.5	Ir-O	5.0 ± 0.6	2.00 ± 0.01			11.8 ± 3.1	0.027	95

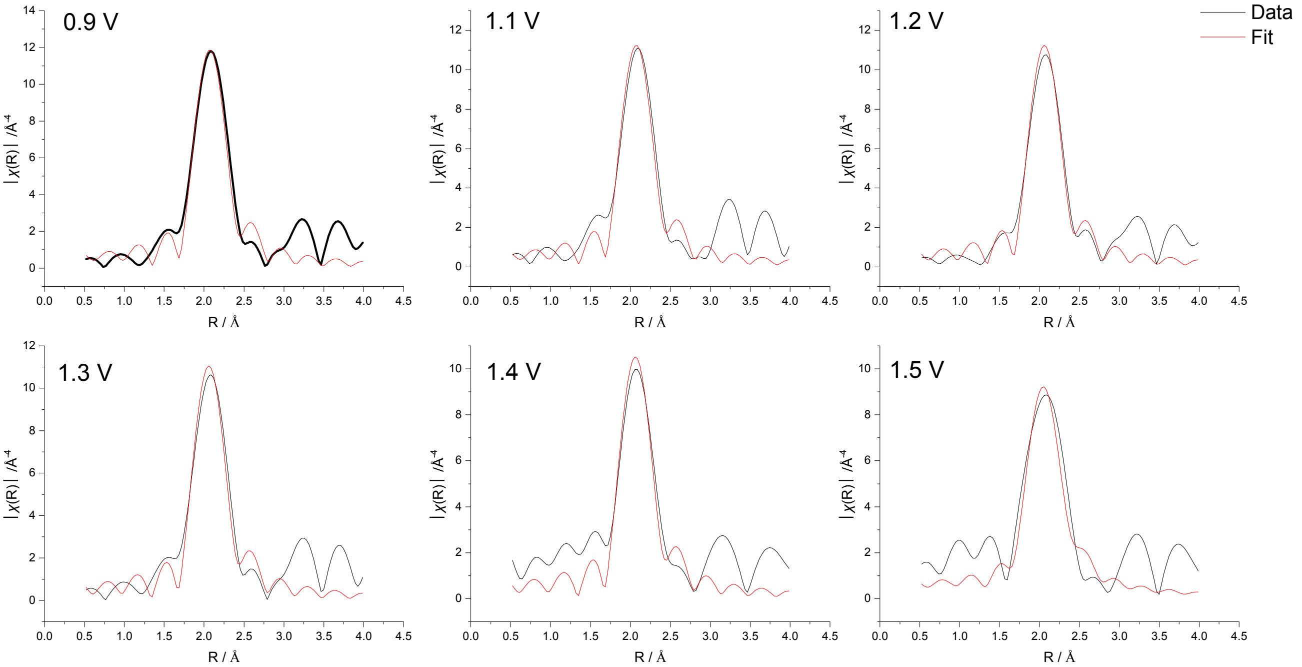

Figure S10 – In situ normalized XANES spectra for a) r-IrO_2, b) IrO_x and c) Li-IrO_x recorded at specified electrode potentials in 0.1M $HClO_4$ on anodic (solid lines) and cathodic sweeps (dotted lines). Insets show an expansion of the absorption peak to highlight the change in position. Fourier transformed Ir EXAFS spectra d) r-IrO_2, e) IrO_x and f) Li-IrO_x at given electrode potentials (k range 3-12).

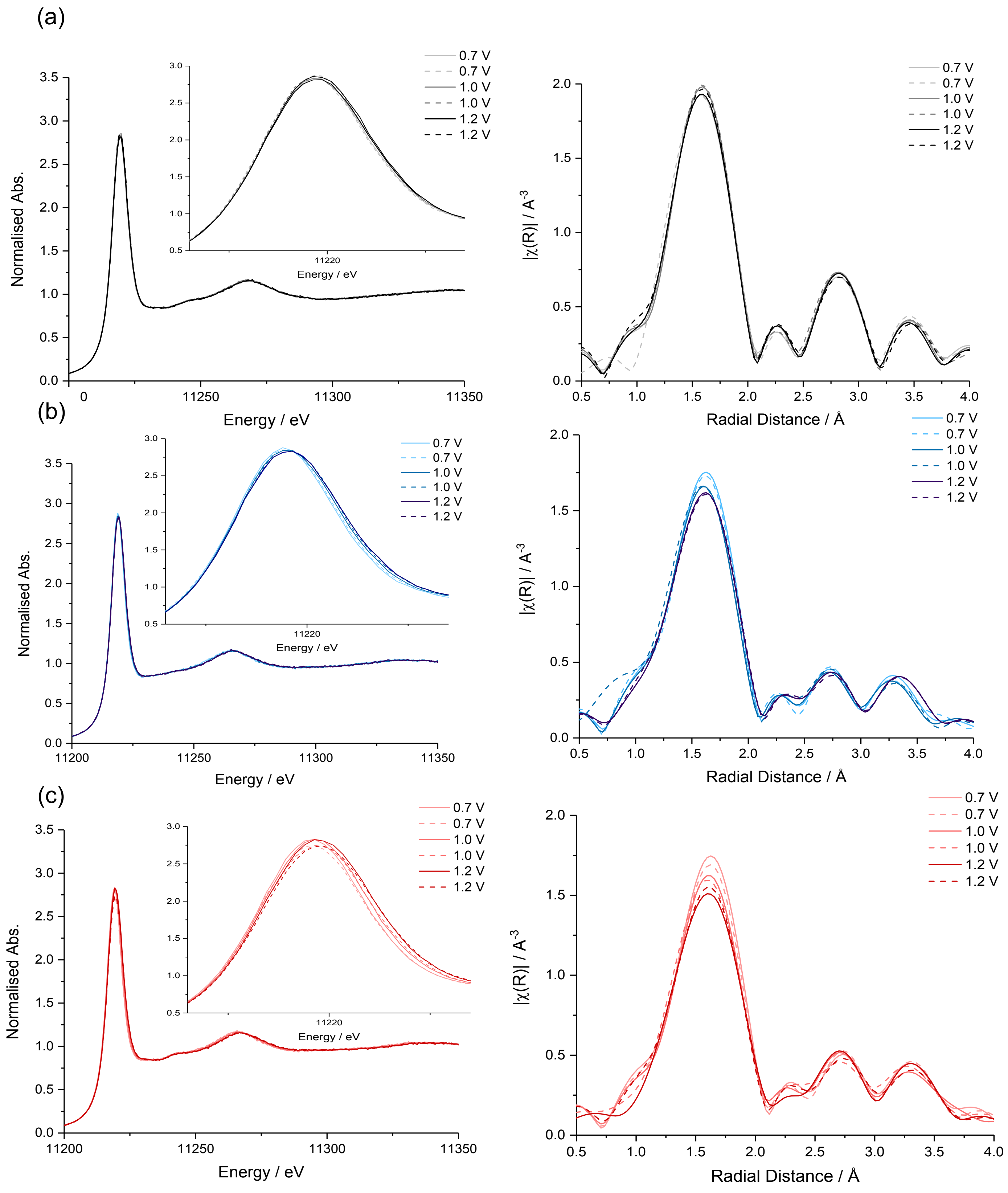

Figure S11 – Correlation between XANES features measured in *opeando* across all materials a) FWHM and WL absorbance b) WL position and WL absorbance and c) FWHM and WL position.

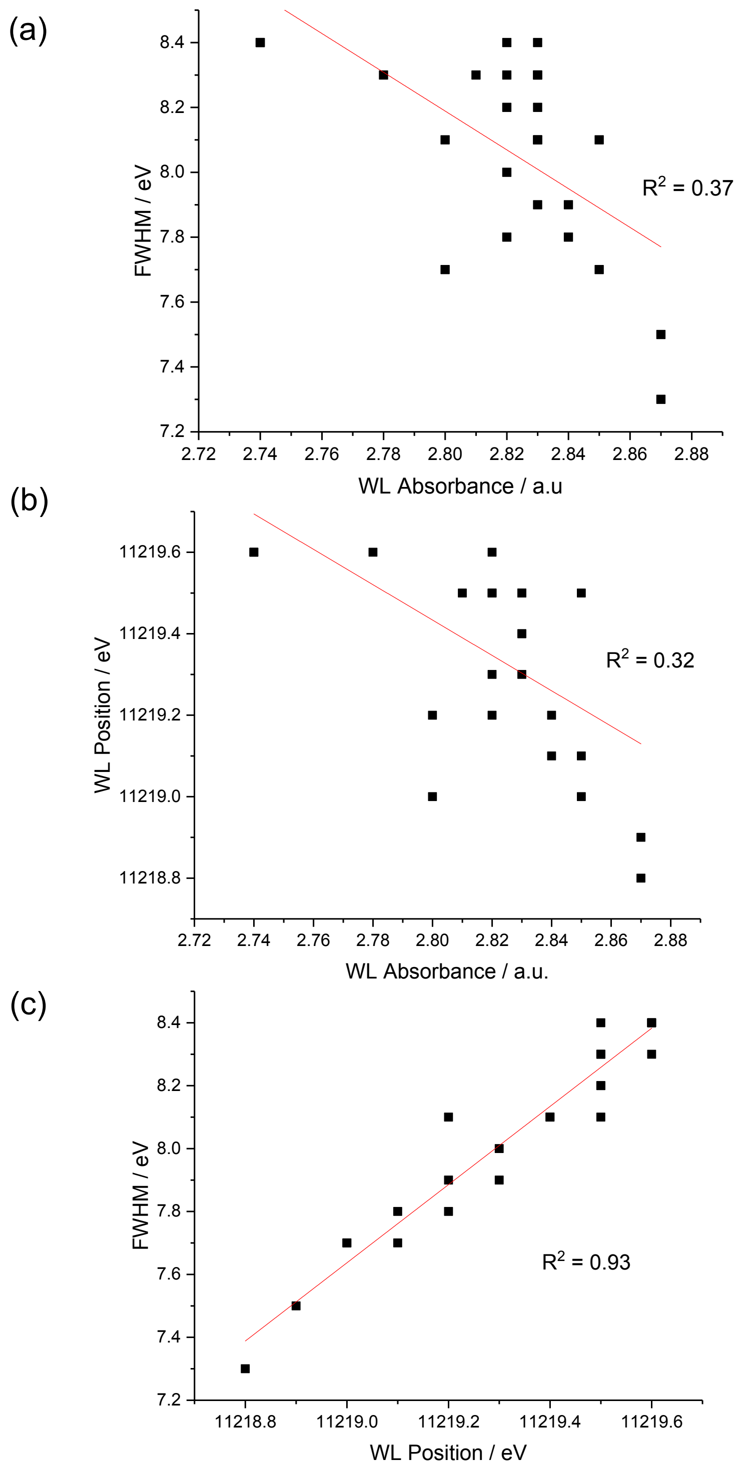

Figure S12: Simulated X-ray absorption spectra in presence of structural defects, oxygen vacancies (left panel) and extra-lattice oxygen (right panel). While both type of localised defects influence intensity, FWHM and asymmetry of the white line, these results suggest that the presence of vacancies has a major impact on the white-line intensity, while the presence of additional O atoms, resulting in a higher oxidation state for Ir, could result in a more pronounced asymmetric profile.

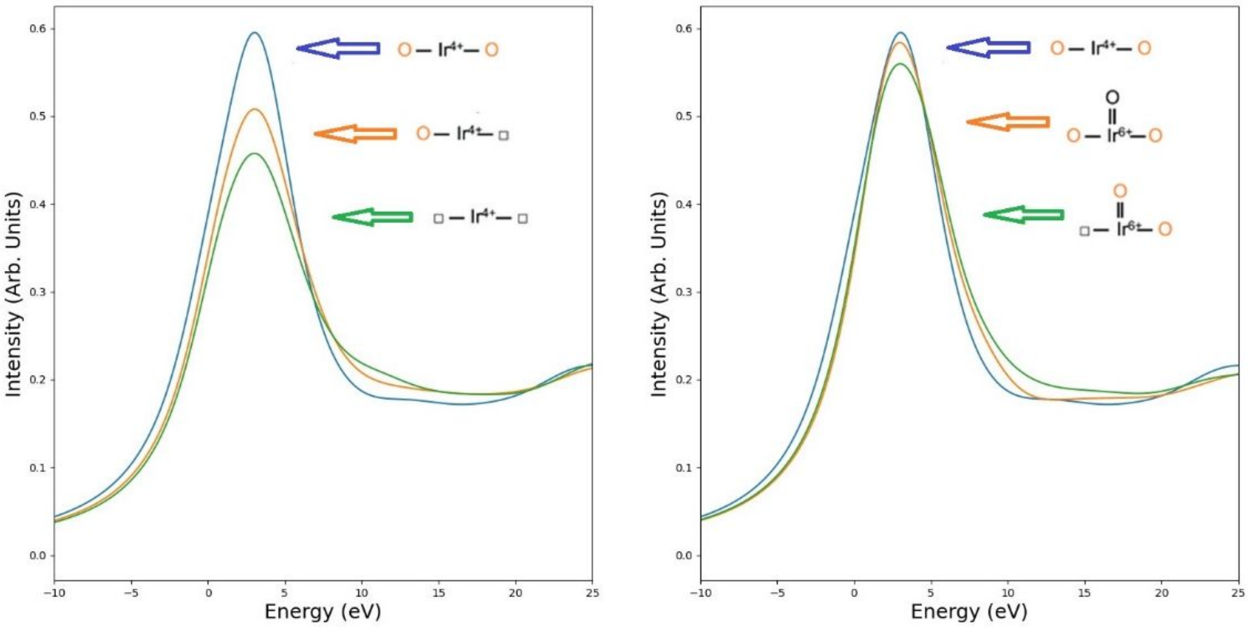

Supplement: Supplementary file 1 — ja2c13567_si_001.pdf [file ja2c13567_si_001.pdf]
